# Endotheliopathy is associated with slower liberation from mechanical ventilation: a cohort study

**DOI:** 10.1186/s13054-021-03877-y

**Published:** 2022-01-30

**Authors:** Martin Schønemann-Lund, Theis S. Itenov, Johan E. Larsson, Birgitte Lindegaard, Pär I. Johansson, Morten H. Bestle

**Affiliations:** 1 Department of Anesthesia and Intensive Care, Copenhagen University Hospital - North Zealand, Copenhagen, Denmark; 2 Department of Pulmonary and Infectious Diseases, Copenhagen University Hospital - North Zealand, Copenhagen, Denmark; 3 Department of Clinical Medicine, University of Copenhagen, Copenhagen, Denmark; 4 Department of Clinical Immunology, Copenhagen University Hospital - Rigshospitalet, Copenhagen, Denmark; 5 Department of Anesthesia and Intensive Care, Copenhagen University Hospital North Zealand, Dyrehavevej 29, 3400 Hillerød, Denmark

**Keywords:** Platelet Endothelial Cell Adhesion Molecule-1, Syndecan-1, Thrombomodulin, Respiratory insufficiency/physiopathology, Endothelium, Vascular, Observational study

## Abstract

**Background:**

Endotheliopathy is suggested as pivotal pathophysiology of sepsis and trauma-associated organ failure, but its role in acute respiratory failure is not yet determined. We investigated if endotheliopathy biomarkers at ICU admission are associated with illness severity and clinical outcomes in patients with acute respiratory failure requiring mechanical ventilation.

**Methods:**

We conducted a prospective single-center cohort study including 459 mechanically ventilated adults at ICU admission. Plasma levels of three endotheliopathy biomarkers were measured at ICU admission: Syndecan-1, soluble Thrombomodulin (sTM), and Platelet Endothelial Cell Adhesion Molecule-1 (PECAM-1). The primary outcome was the rate of liberation from mechanical ventilation, which is presented together with the rate of the competing risk of death while still on mechanical ventilation. Secondary outcomes were PaO_2_/FiO_2_-ratios on admission and on last measurement in patients dying within five days, and 30-day all-cause mortality. The primary outcome and 30-day all-cause mortality were analyzed using Cox regression, controlled for gender, age, chronic obstructive pulmonary disease, septic shock, heart failure, PaO_2_/FiO_2_-ratio at admission, respiratory infection, acute kidney injury, and bilirubin. PaO_2_/FiO_2_-ratios were analyzed using linear regression, controlled for age, chronic obstructive pulmonary disease, respiratory infection, and shock.

**Results:**

Patients with high sTM were liberated from mechanical ventilation at a lower rate (adjusted hazard ratio (HR) 0.71, for an increase from the 25th to the 75th percentile, 95% confidence interval (CI) 0.54–0.93, *p* = 0.01). Patients with high PECAM-1 were liberated from mechanical ventilation at a lower rate, but only during the first 5 days (adjusted HR 0.72, for an increase from the 25th to the 75th percentile, 95% CI 0.58–0.9, *p* < 0.01). High levels of Syndecan-1 and PECAM-1 were associated with a higher rate of death while still on mechanical ventilation. sTM and PECAM-1 were negatively associated with PaO_2_/FiO_2_-ratio at ICU admission and no biomarker was associated with last measured PaO_2_/FiO_2_-ratio. High levels of all biomarkers were associated with higher 30-day all-cause mortality.

**Conclusion:**

In acute respiratory failure, endotheliopathy biomarkers are associated with lower rates of liberation from mechanical ventilation, hypoxemia at ICU admission, and 30-day all-cause mortality.

**Graphic Abstract:**

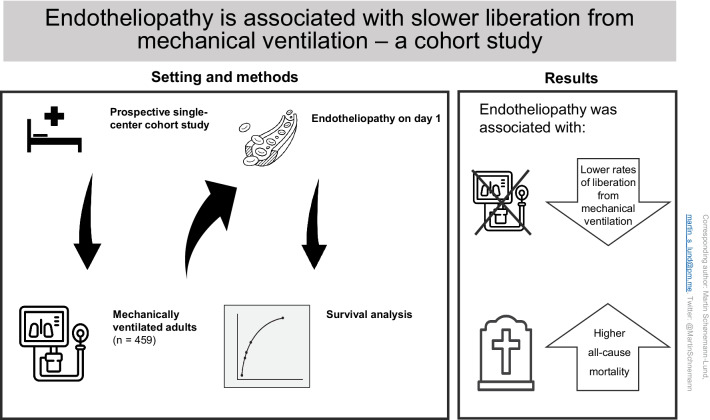

**Supplementary Information:**

The online version contains supplementary material available at 10.1186/s13054-021-03877-y.

## Background

Acute respiratory failure requiring mechanical ventilation (MV) is a common cause of admission to intensive care units (ICUs), and worldwide up to 20 million people are mechanically ventilated per year [1]. Survival from acute respiratory failure has increased over time, but mortality remains high, estimated at 27–35% [2–5].

To further the pathophysiological understanding, aid in detection of at-risk patients, and find potential new treatment targets in acute respiratory failure, a host of biomarkers have been suggested for use [6, 7], but none has made it into routine clinical practice.

Endotheliopathy may be unifying pathophysiology in acute, critical illness, linking different primary pathologies with the common outcome of multiple organ failure [8–10]. Endothelial cells and their luminal coating—the glycocalyx—are crucial for maintaining vascular membrane integrity [11], and one of the defining features of Acute Respiratory Distress Syndrome (ARDS) is fluid leak from the alveolar capillaries [12].

Syndecan-1, a proteoglycan part of the endothelial glycocalyx [11, 13], is one of the most studied biomarkers of glycocalyx shedding [11], and levels in blood are increased in multiple disease states [13].

Thrombomodulin is a transmembrane protein located on the luminal surface of endothelial cells [14]. It is involved in the activation of protein C and exerts important anticoagulant and anti-inflammatory actions [15, 16]. In plasma, Thrombomodulin is found in a soluble form (sTM) [17] that is released in vitro in response to endothelial cell damage [18] and used as a marker of direct cell damage [19].

Platelet Endothelial Cell Adhesion Molecule-1 (PECAM-1) is a member of the Ig-superfamily. It forms part of the tight junctions between endothelial cells [20, 21] and is crucial for maintaining the structural integrity of the endothelium [22].

Together, Syndecan-1, sTM, and PECAM-1 reflect three important aspects of endotheliopathy: shedding of the endothelial glycocalyx (Syndecan-1); direct damage to the endothelial cells (sTM); and disruption of the tight junctions between the endothelial cells (PECAM-1). Syndecan-1 and sTM—alone and in conjunction—have been associated with the risk of developing acute respiratory failure as well as outcomes of manifest respiratory failure [23–29]. However, those results are not unanimous [19, 30], and PECAM-1 has not previously been included in studies of endotheliopathy in acute respiratory failure.

We hypothesized that endotheliopathy is an important part of the pathophysiology of acute respiratory failure. To find out, we assessed the association of endotheliopathy, as reflected by plasma levels of Syndecan-1, sTM, and PECAM-1, with clinical outcomes and disease severity in acute respiratory failure requiring MV in the ICU.

## Methods

### Study design, setting, and participants

The present study is reported in accordance with the STROBE guidelines [31].

The Metabolomics study was a prospective, single-center cohort study investigating the role of endothelial metabolomics in critically ill patients.

We included patients from the Metabolomics study receiving invasive or non-invasive (NIV) positive pressure ventilation on the first day of their ICU stay.

Recruitment was between November 2016 and June 2019 in the ICU at Copenhagen University Hospital—North Zealand, a 12-bed, surgical/medical ICU with approximately 900 admissions/year.

Patients aged 18 years or more, acutely admitted to the ICU with an expected stay > 24 h were eligible. Exclusion criteria were non-obtainable informed consent or if active treatment was deemed futile by the treating clinician.

Included patients had study blood samples drawn daily during the first five days of the ICU stay. For the present study, the first obtained samples were analyzed.

Patient participation was initially approved by a trial guardian. Relatives and/or patients were subsequently approached for informed consent. The local ethical committee approved the study (H-17027963) as did the Danish Data Protection Agency (I-suite nr.: 04673 and 04674).

### Outcomes

The primary outcome was liberation from MV within 30 days from study inclusion, defined to occur on the second of two consecutive days without the need for either invasive or non-invasive positive pressure ventilation. Right-censoring occurred when patients were either transferred to a non-study ICU or reached 30 days from inclusion while still mechanically ventilated.

Prespecified secondary outcomes were 30-day all-cause mortality, worst PAF-ratio on the first day of ICU-admission, and last PAF-ratio measured in patients dying within five days from inclusion. Post-hoc we performed additional exploratory analyses using as outcomes: worst oxygenation index (OI) [32] on the first day of ICU-admission; worst ventilatory ratio (VR) [33] on the first day of ICU-admission; last OI measured in patients dying within five days from inclusion; and last VR measured in patients dying within five days from inclusion.

### Measurements

EDTA-plasma was separated after centrifuging at 3000 rounds per minute for 10 min directly after collection and stored at − 80 °C. Samples underwent one freeze–thaw cycle for analysis of Syndecan-1, sTM, and PECAM-1 consecutively, for a total of 3 freeze–thaw cycles. Syndecan-1, sTM, and PECAM-1 were analyzed in uniplicate using enzyme-linked immunosorbent assays (Syndecan-1 & sTM: Diaclone SAS, Besancon, France; PECAM-1: R&D System, Minneapolis, USA). The lower limits of detection were 4.94 ng/ml (Syndecan-1), 0.31 ng/ml (sTM) and 0.021 ng/ml (PECAM-1). The intra- and inter-assay coefficients of variation were 6.2% and 10.2% (Syndecan-1), 3.9% and 9.8% (sTM) and 3.3% and 6.9% (PECAM-1), respectively.

### Statistical analysis

We developed a statistical analysis plan and published it on our institution’s website before undertaking the final analyses. The analysis plan is available in the online supplementary material, including details on statistical methods and definitions of variables and outcomes (Additional File 1).

We analyzed the association between the endotheliopathy biomarkers and the primary outcome as well as all-cause mortality using Cox regression. For each outcome investigated, separate models were fitted for each of the three biomarkers. The endotheliopathy biomarkers were introduced to the statistical models in two different ways: 1) as continuous variables; 2) as categorical variables divided into three groups (1st quartile, 2nd & 3rd quartile, 4th quartile). Models were controlled for sex, age, history of chronic obstructive pulmonary disease (COPD), septic shock, history of heart failure, initial PAF-ratio, Respiratory Infection, acute kidney injury, and bilirubin. Post-hoc we fitted models for the primary outcome substituting initial PAF-ratio with OI or VR. We also fitted models for the primary outcome substituting all confounders with the full SAPS 3 score.

For the primary outcome, we report cause-specific hazard ratios (HR) with 95% confidence intervals (95% CI) along with those for the competing risk of death while still on MV. Specifically, we treated the event of death while still on MV as right-censoring in the estimation of the HR for liberation from MV and vice versa [34]. Post-hoc we decided to also report the 30-day absolute risk of liberation from MV and death while still on MV obtained with the method by Ozenne et al. [34].

For models with the endotheliopathy biomarkers introduced as continuous variables, we report the HR associated with an increase from the 25th percentile to the 75th percentile. This is to aid understanding, as HRs associated with a 1 ng/ml increase in biomarkers are usually small and difficult to interpret. For models with the endotheliopathy biomarkers introduced as categorical variables, we report the HRs associated with each group, using 1st quartile as reference.

We tested if the associations of the endotheliopathy biomarkers with the primary outcome and with 30-day all-cause mortality were different in different subgroups. We prespecified testing if the associations were different in patients with or without septic shock at baseline or in patients with or without respiratory infection at baseline. Post-hoc, we added the following subgroup analyses: 1) the type of mechanical ventilation (invasive or non-invasive) at baseline; 2) a known history of COPD; 3) the type of admission (medical or surgical).

To explore the discriminatory abilities of the endotheliopathy biomarkers further, we post hoc performed a Receiver Operating Characteristics (ROC)-curve-analysis for predicting both the primary outcome and 30-day all-cause mortality, including baseline levels of C-reactive protein (CRP) as a reference.

The associations with PAF-ratio, OI, and VR were analyzed using multiple linear regression. Models were controlled for age, COPD, respiratory infection, and shock (defined as receiving vasopressor plus blood lactate > 2 regardless of the cause).

Counts (%) and medians (interquartile range [IQR]) are presented as appropriate. Statistical significance was set at *p* < 0.05. All analyses were performed with R, version 3.6.1 [35].

## Results

**Fig. 1:**
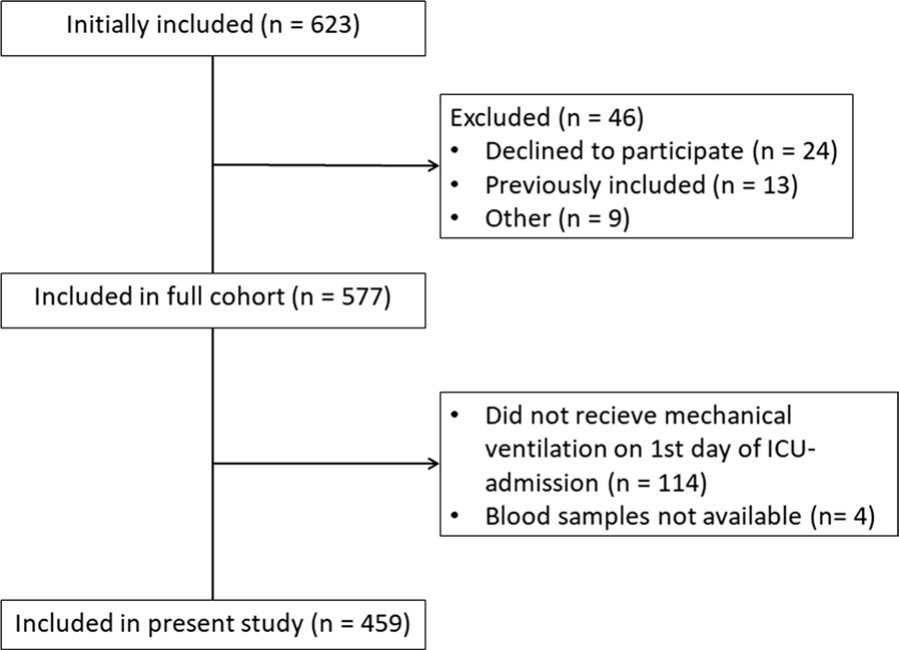
Flowchart of the inclusion/exclusion-process

Out of the 577 patients in the Metabolomics cohort, 459 patients were included in the study presented here (Fig. 1). The median age was 71 years (IQR 63–79 years), 40.7% were female, 24.0% had diabetes, 53.2% had arterial hypertension, 33.6% had COPD, and the median body mass index was 25.6 (IQR 22.9–30.5) (Table 1). All patients were critically ill with a median simplified acute physiology score 3 (SAPS 3) of 65 (IQR 56–75). 95% of patients presented with failure of more than one organ as defined by a Sequential Organ Failure Assessment (SOFA) subscore of ≥ 2 in > 1 of the respiratory, circulatory, central nervous system, kidney, or liver sub-scores (Table 1). 30.7% received NIV and 69.3 received invasive ventilation at baseline. The main reasons for MV were COPD (20.3%), non-pulmonary sepsis (21.4%), or pneumonia (20.3%) (Table 1). The median levels were 45.3 ng/ml (IQR 21.0–118.4 ng/ml) for Syndecan-1, 10.1 ng/ml (IQR 5.0–18.0 ng/ml) for sTM, and 13.1 ng/ml (11.1–14.6 ng/ml) for PECAM-1. Levels of sTM and PECAM were higher among patients with diabetes and chronic kidney disease than among patients without, patients with chronic heart failure had higher levels of PECAM-1 than patients without, whereas patients without COPD and hypertension had lower values of sTM and Syndecan-1, respectively (Additional File 2: Table S1). Patient treatment during the first five days in the ICU is summarized in Additional File 2: Table S2.

**Table 1:** Patient characteristics at baseline

Variables	Levels	All patients (*n* = 459)
Age, years	Median (IQR)	71.0 (63.0–79.0)
Sex, female	*n* (%)	187 (40.7)
Body mass index, kg/m^2^	Median (IQR)	25.7 (23.0–30.9)
History of stroke	*n* (%)	50 (10.9)
History of COPD	*n* (%)	154 (33.6)
History of hypertension	*n* (%)	244 (53.2)
History of myocardial infarction	*n* (%)	54 (11.8)
History of heart failure	*n* (%)	34 (7.4)
History of diabetes	*n* (%)	110 (24.0)
Type of admission		
Medical	*n* (%)	356 (77.6)
Surgical	*n* (%)	103 (22.4)
Mechanical ventilation at baseline		
NIV	*n* (%)	141 (30.7)
Invasive	*n* (%)	318 (69.3)
Primary reason for mechanical ventilation		
Aspiration	*n* (%)	22 (4.8)
Cardiac arrest	*n* (%)	31 (6.8)
COPD	*n* (%)	93 (20.3)
Intoxication	*n* (%)	14 (3.1)
Neurological disease	*n* (%)	36 (7.8)
Non-pulmonary sepsis	*n* (%)	98 (21.4)
Other cause of acute respiratory failure	*n* (%)	29 (6.3)
Pneumonia	*n* (%)	93 (20.3)
Postoperative	*n* (%)	43 (9.4)
Respiratory infection	*n* (%)	214 (46.6)
PaO_2_/FiO_2_-ratio, kPa	Median (IQR)	21.4 (15.0–28.0)
Oxygenation index	Median (IQR)	5.8 (3.9–10.1)
Ventilatory ratio	Median (IQR)	1.6 (1.2–2.0)
Bilirubin, µmol/L	Median (IQR)	10.7 (7.2–17.7)
KDIGO Score ≥ 2	*n* (%)	115 (25.1)
Septic shock	*n* (%)	85 (18.5)
Multi-organ failure^a^	*n* (%)	436 (95.0)
SAPS 3	Median (IQR)	65.0 (56.0–75.0)
C-reactive protein, mg/L	Median (IQR)	93.1 (30.9–179.9)
Syndecan-1, ng/ml	Median (IQR)	45.3 (21.0–118.4)
sTM, ng/ml	Median (IQR)	10.1 (5.0–18.0)
PECAM-1, ng/ml	Median (IQR)	13.1 (11.1–14.6)

*COPD* chronic obstructive pulmonary disease, *SAPS 3* Simplified Acute Physiology Score 3, *KDIGO-score* kidney disease, Improving Global Outcomes-score, *NIV* non-invasive ventilation, *sTM* Soluble Thrombomodulin, *PECAM-1* Platelet Endothelial Cell Adhesion Molecule-1, *IQR* Interquartile Range

^a^Defined as a Sequential Organ Failure sub score of 2 or more in more than 1 of the respiratory, circulatory, central nervous system, kidney or liver sub scores

### Liberation from mechanical ventilation

**Fig. 2:**
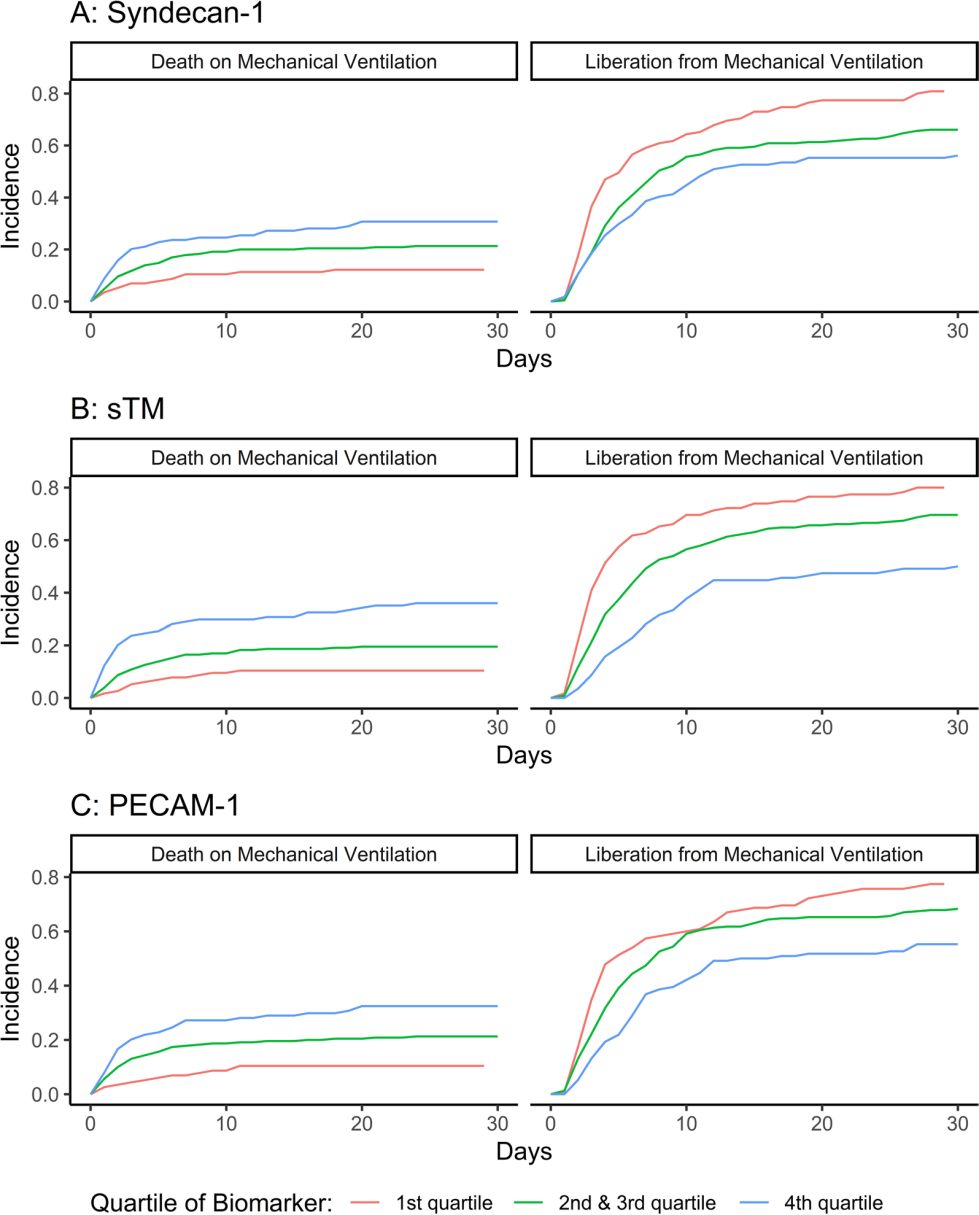
Cumulative incidences of liberation from mechanical ventilation and the competing risk of death on mechanical ventilation, divided on the grouped versions of Syndecan-1, soluble Thrombomodulin (sTM) and Platelet Endothelial Cell Adhesion Molecule-1 (PECAM-1) respectively

After 30 days, 309 patients were liberated from MV, 98 died while on MV, and 52 patients were censored. Of the latter group, 18 reached 30 days from inclusion while still on MV, and 34 were transferred to non-study ICUs, mainly due to ICU capacity issues. Of those liberated from MV, 13 were reintubated and 15 were treated with NIV during follow-up. Cumulative incidence curves for the primary outcome are summarized in Fig. 2. Survivors and non-survivors were mechanically ventilated for a median of 4.6 (IQR 2.6–9.6) and 2.9 (IQR 1.4–6.4) days, respectively.

**Fig. 3:**
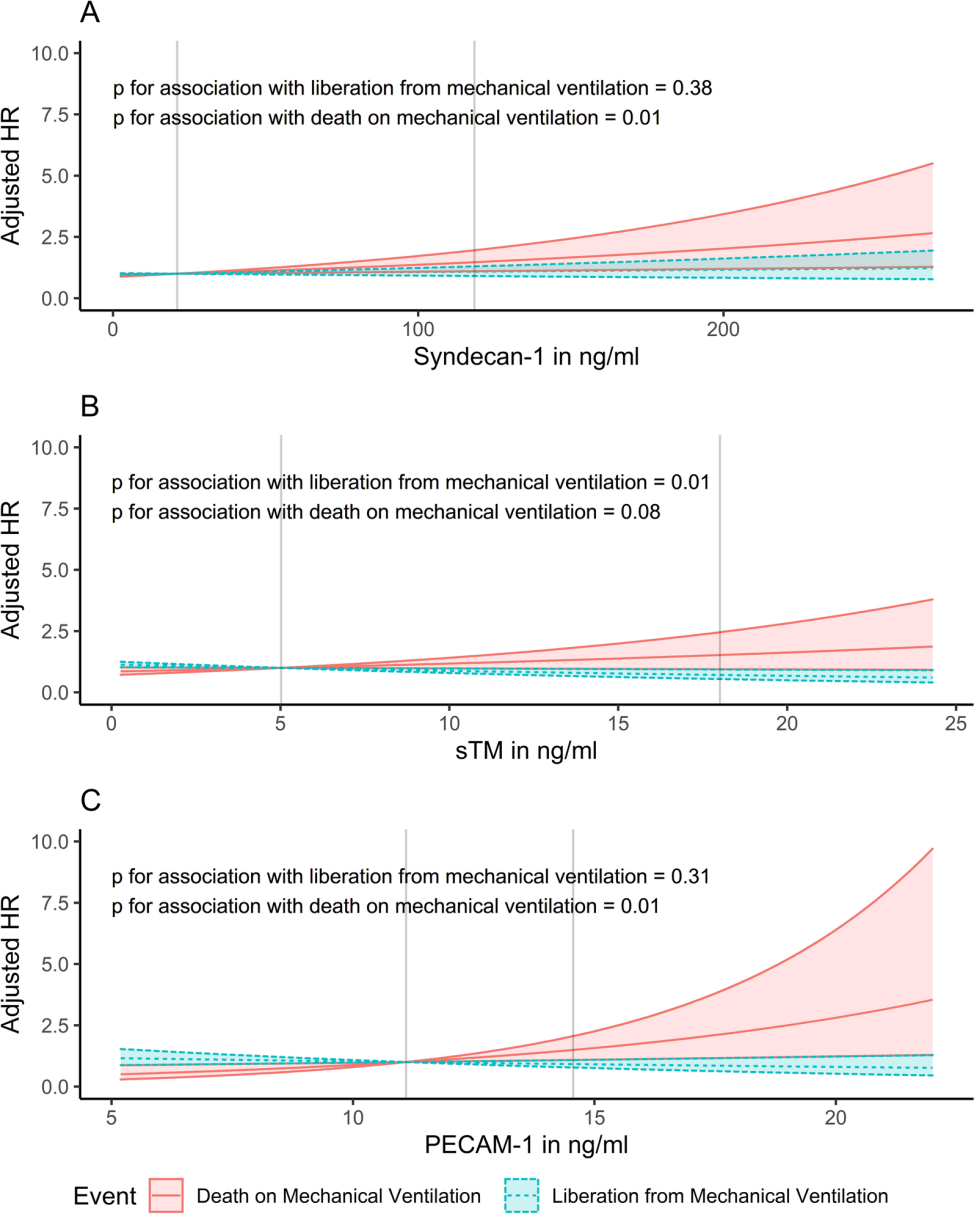
Adjusted hazard ratios (HRs) for liberation from mechanical ventilation and the competing risk of death on mechanical ventilation with point estimates and 95% confidence intervals against the levels of Syndecan-1, soluble Thrombomodulin (sTM) and Platelet Endothelial Cell Adhesion Molecule-1 (PECAM-1) respectively. The HRs are adjusted for age, gender, chronic obstructive pulmonary disease, heart failure, bilirubin, PaO_2_/FiO_2_-ratio, septic shock and respiratory infection. Vertical lines inserted at the 25th and 75th percentiles of each biomarker for illustrative purposes

**Fig. 4:**
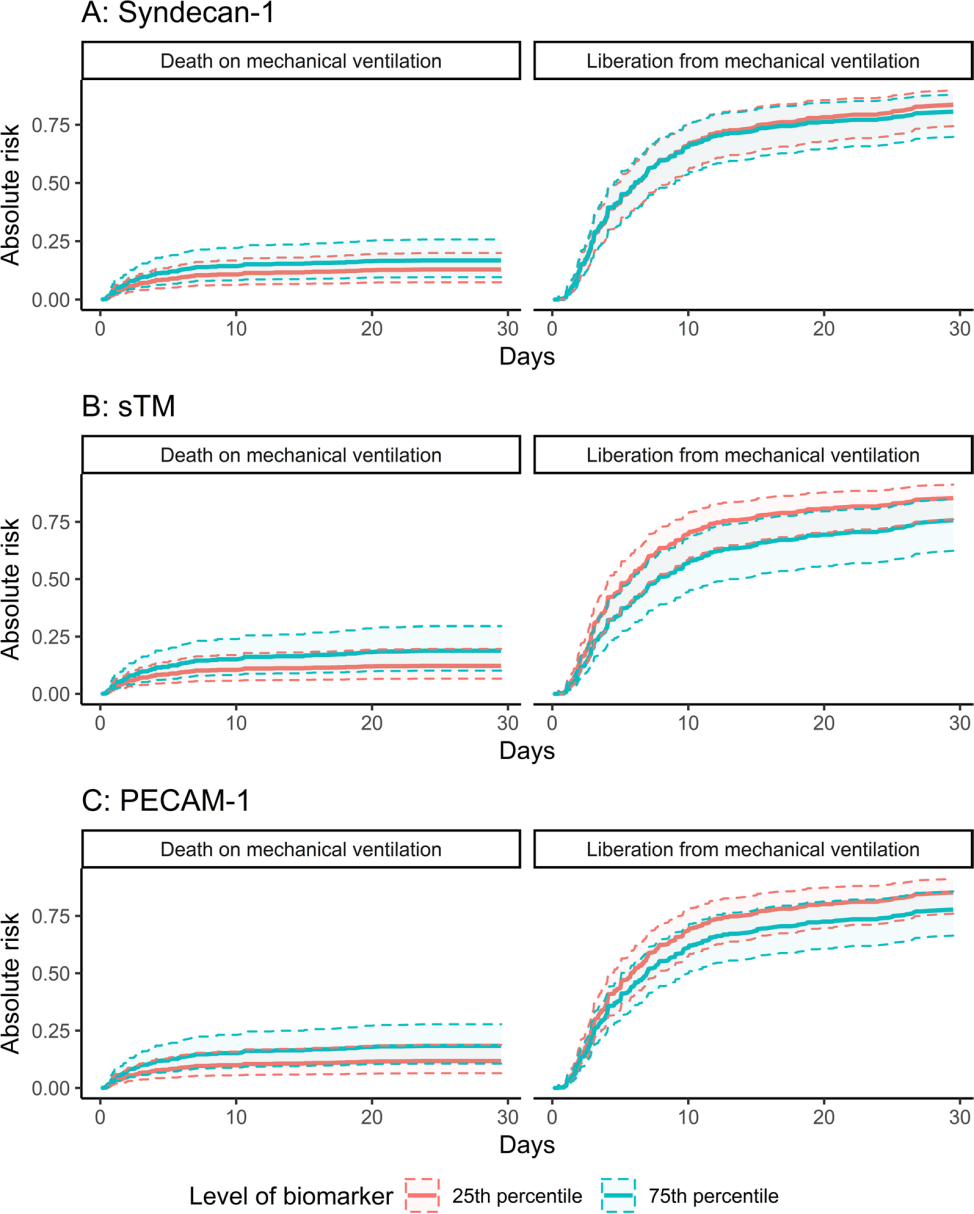
Absolute risks of liberation from mechanical ventilation and of death on mechanical ventilation comparing patients with levels at the 25th percentile with patients with levels at the 75th percentile of Syndecan-1, Soluble Thrombomodulin (sTM) and Platelet Endothelial Cell Adhesion Molecule-1 (PECAM-1) respectively. Solid lines are point estimates and dashed lines represent 95% confidence intervals. The absolute risks are calculated based on the cause specific hazard rates for the represented outcomes, obtained from multivariable competing risks Cox regressions introducing Syndecan-1, sTM or PECAM-1 as continuous variables. The absolute risks are calculated for a 71-year old female, with no chronic obstructive pulmonary disease, no chronic heart failure, no respiratory infection at baseline, no acute kidney injury at baseline, with a Bilirubin of 10.7 µmol/L and a PaO_2_/FiO_2_-ratio of 21.4 kPa

When analyzing the endotheliopathy biomarkers as continuous variables, we found that patients with higher levels of Syndecan-1 (HR 0.84 for an increase from the 25th to the 75th percentile [95% CI 0.72–0.99, *p* = 0.03]), sTM (HR 0.57 for an increase from the 25th to the 75th percentile [95% CI 0.46–0.72, *p* < 0.01]) and PECAM-1 (HR 0.8 for an increase from the 25th to the 75th percentile [95% CI 0.69–0.93, *p* < 0.01]) had lower rates of liberation from MV before adjustment. After adjustment, only patients with higher levels of sTM (HR 0.71 for increase from the 25th to the 75th percentile [95% CI 0.54–0.93, *p* = 0.01]) had lower rates of liberation from MV (Additional File 2: Tables S3–S5; Fig. 3). Patients with higher levels of Syndecan-1 and PECAM-1 had higher rates of dying while mechanically ventilated in both the unadjusted and adjusted analyses, whereas sTM was not associated with this outcome after adjustment (Additional File 2: Tables S3–S5; Fig. 3). From the obtained cause-specific HRs for liberation from MV and death while still on MV, absolute risks for both events were calculated. The absolute 30-day risk of liberation from MV and of death while still on MV comparing the 25th percentile with the 75th percentile of the endotheliopathy biomarkers are found in Table 2 and graphically represented in Fig. 4.

**Table 2:** 30-day absolute risk of liberation from mechanical ventilation and of the competing risk of death on mechanical ventilation in mechanically ventilated patients in the intensive care unit—results from competing risks Cox regression (*n* = 459)

	30-day absolute risk (95% CI)^a^			
	Liberation from mechanical ventilation		Death on mechanical ventilation	
Biomarker^†^	25th percentile	75th percentile	25th percentile	75th percentile
Syndecan-1	0.84 (0.74–0.9)	0.81 (0.7–0.88)	0.13 (0.07–0.2)	0.17 (0.1–0.26)
sTM	0.85 (0.76–0.91)	0.76 (0.62–0.85)	0.12 (0.07–0.2)	0.19 (0.1–0.29)
PECAM-1	0.85 (0.76–0.91)	0.78 (0.66–0.86)	0.12 (0.06–0.19)	0.18 (0.11–0.28)

^a^The absolute risks are calculated for a 71-year old female, with no chronic obstructive pulmonary disease, no chronic heart failure, no respiratory infection at baseline, no acute kidney injury at baseline, with a Bilirubin of 10.7 µmol/L at baseline and a PaO_2_/FiO_2_-ratio of 21.4 kPa at baseline

^†^Biomarkers were analyzed as continuous variables and the absolute risks associated with values at the 25th and the 75th percentile are presented: 21.0 and 118.4 ng/ml (Syndecan-1), 5.0 and 18.0 ng/ml (soluble Thrombomodulin [sTM]), 11.1 and 14.6 ng/ml (Platelet Endothelial Cell Adhesion Molecule-1 [PECAM-1]). *95% CI* 95% confidence interval

Additional modeling substituting PAF-ratio with VR or OI and all confounders with SAPS 3 gave confirming results, except that the association of sTM with liberation from MV did not reach significance in the model controlled for OI (Additional File 2: Tables S6–S14).

There was no evidence of difference in associations between the endotheliopathy biomarkers and the primary outcome in patients with or without septic shock or respiratory infection (Additional File 3–5: Figs. S1–S3). We additionally tested the associations between the endotheliopathy biomarkers and the primary outcome in patients with or without COPD, in patients receiving NIV or invasive ventilation, and in patients admitted for surgical or medical reasons. We found that COPD patients with higher levels of Syndecan-1 had higher rates of liberation from MV and that patients receiving NIV with higher levels of PECAM-1 had lower rates of liberation from MV (Additional File 3–5: Figs. S1–S3).

**Fig. 5:**
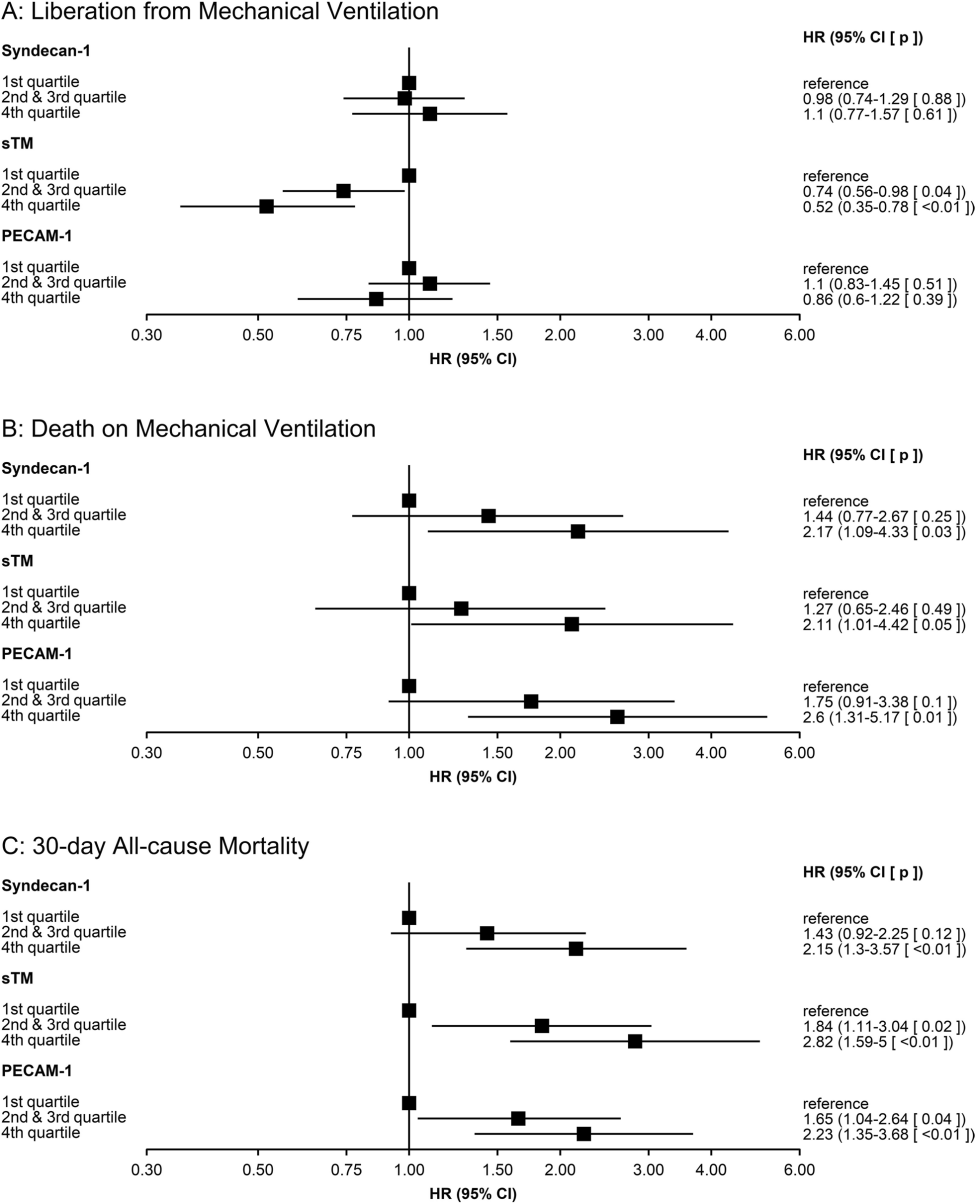
Plotted associations of Syndecan-1, soluble Thrombomodulin (sTM) and Platelet Endothelial Cell Adhesion Molecule-1 (PECAM-1) as categorical variables. 1st quartile is used as reference. The HRs are adjusted for age, gender, chronic obstructive pulmonary disease, heart failure, bilirubin, PaO_2_/FiO_2_-ratio, septic shock and respiratory infection. *HR* hazard ratio, *95% CI* 95% confidence interval

When analyzing the three biomarkers as categorical variables, we found that higher levels of sTM were significantly associated with a lower rate of liberation from MV after adjustment (HR 0.52 for the 4th vs. the 1st quartile [95% CI 0.35–0.78, *p* < 0.01]; Fig. 5). All three biomarkers were significantly associated with a higher rate of death on MV when comparing the 4th with the 1st quartile (Fig. 5).

From inspection of Fig. 2 as well as formal testing of the assumption of proportional hazards, we suspected that the effect of sTM and PECAM-1 on the rate of liberation from MV varied over time, having a larger impact in the first 5 days during which nearly 2/3 of the events took place. We, therefore, separated the dataset in time intervals: day 1–5, day > 5–12, and > day 12. We then conducted an analysis allowing the estimates for the effect of Syndecan-1, sTM, and PECAM-1 to vary between different time intervals.

For Syndecan-1 and sTM this analysis largely confirmed the results from the main analysis. For PECAM-1, however, there was a marked association with the rate of liberation from MV during the first 5 days (HR 0.72 for an increase from the 25th to the 75th percentile [95% CI 0.58–0.9, *p* < 0.01]) (Table 3). In the > 5 to 12-day period, patients with higher levels of PECAM-1 instead had higher rates of liberation from MV (Table 3).

**Table 3:** Time-varying associations of Syndecan-1, soluble Thrombomodulin (sTM) and Platelet Endothelial Cell Adhesion Molecule-1 (PECAM-1) with liberation from mechanical ventilation and the competing risk of death on mechanical ventilation in mechanically ventilated patients in the intensive care unit—Cox regression (*n* = 459)

Biomarker^†^	Multivariable^a^			
	Liberation from mechanical ventilation		Death on mechanical ventilation	
	Hazard ratio (95% CI)	*p*	Hazard ratio (95% CI)	*p*
Syndecan-1, 25th versus 75th percentile, 5 days	1.01 (0.8–1.28)	0.92	1.56 (1.12–2.18)	**0.01**
Syndecan-1, 25th versus 75th percentile, > 5–12 days	1.42 (1.07–1.88)	**0.01**	0.64 (0.3–1.36)	0.24
Syndecan-1, 25th versus 75th percentile, > 12 days	0.72 (0.4–1.27)	0.25	2.87 (1.26–6.5)	**0.01**
sTM, 25th versus 75th percentile, 5 days	0.58 (0.41–0.81)	** < 0.01**	1.6 (0.92–2.77)	0.09
sTM, 25th versus 75th percentile, > 5–12 days	1.26 (0.84–1.91)	0.26	0.51 (0.2–1.32)	0.16
sTM, 25th versus 75th percentile, > 12 days	0.41 (0.21–0.81)	**0.01**	13.06 (1.52–112.44)	**0.02**
PECAM-1, 25th versus 75th percentile, 5 days	0.72 (0.58–0.9)	** < 0.01**	1.49 (1.01–2.2)	**0.04**
PECAM-1, 25th versus 75th percentile, > 5–12 days	1.67 (1.25–2.23)	** < 0.01**	0.92 (0.47–1.8)	0.8
PECAM-1, 25th versus 75th percentile, > 12 days	0.6 (0.39–0.93)	**0.02**	3.42 (1.25–9.34)	**0.02**

*P*-values < 0.05 are marked with bold

^a^The multivariable analyses were controlled for gender, age, history of chronic obstructive pulmonary disease, septic shock, history of heart failure, initial PaO_2_/FiO_2_-ratio, respiratory Infection, acute kidney injury and bilirubin, ^†^biomarkers were analyzed as continuous variables and the hazard ratios associated with an increase from 25th to the 75th percentile are presented: 21.0–118.4 ng/ml (Syndecan-1), 5.0–18.0 ng/ml (sTM) and 11.1–14.6 ng/ml (PECAM-1). *95% CI* 95% confidence interval

ROC-curve-analysis showed similar performance among the endotheliopathy biomarkers and CRP for predicting liberation from MV, with Area Under the Curve (AUC) ranging from 0.6 (CRP) to 0.66 (sTM) (Additional File 6: Fig. S4). For predicting death while still on MV, sTM showed superior performance compared to CRP (AUC 0.65 vs. 0.54, *p* = 0.01), all other comparisons were non-significant (Additional File 7: Fig. S5).

### 30-day all-cause mortality

**Fig. 6:**
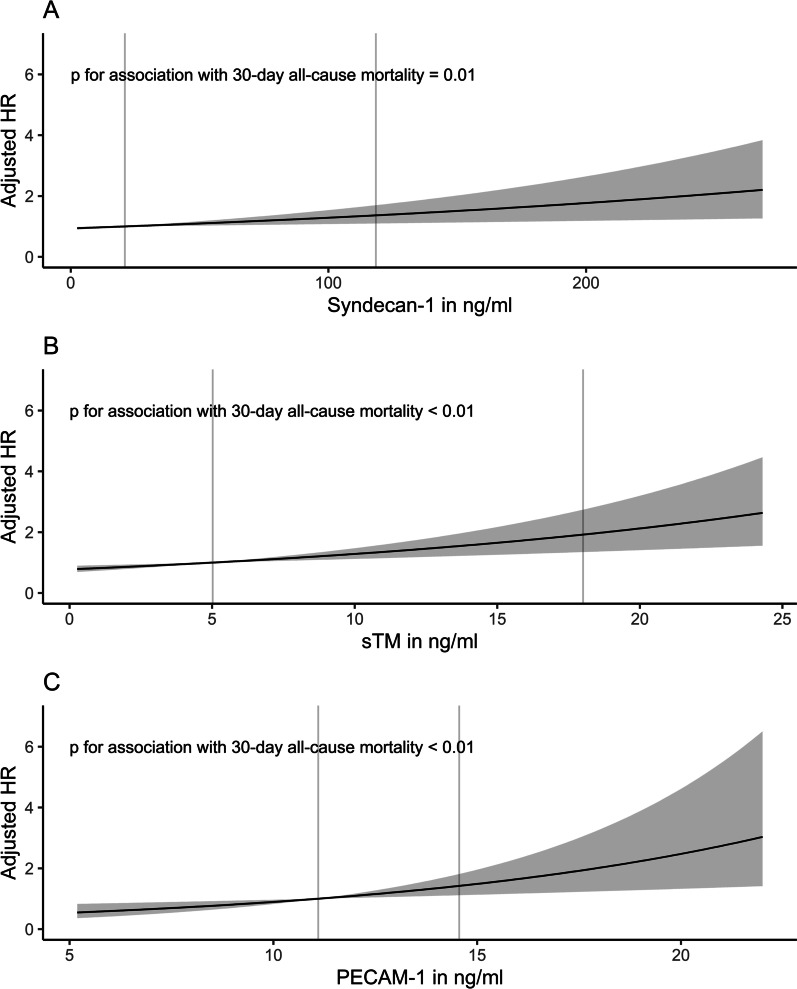
Adjusted hazard ratios (HRs) for 30-day all-cause mortality with point estimates and 95% confidence intervals against the levels of Syndecan-1, soluble Thrombomodulin (sTM) and Platelet Endothelial Cell Adhesion Molecule-1 (PECAM-1) respectively. The HRs are adjusted for age, gender, chronic obstructive pulmonary disease, heart failure, bilirubin, PaO_2_/FiO_2_-ratio, septic shock and respiratory infection. Vertical lines inserted at the 25th and 75th percentiles of each biomarker for illustrative purposes

After 30 days, a total of 163 patients had died. Follow-up for deaths was complete. When analyzed as continuous variables and after adjustment, patients with higher levels of Syndecan-1 (HR 1.36 for an increase from the 25th to the 75th percentile [95% CI 1.1–1.7, *p* < 0.01]), sTM (HR 1.92 for an increase from the 25th to the 75th percentile [95% CI 1.35–2.74, *p* < 0.01]) and PECAM-1 (HR 1.42 for an increase from the 25th to the 75th percentile [95% CI 1.12–1.81, *p* = 0.01]) had higher rates of death (Additional File 2: Tables S15–S17; Fig. 6). There was no evidence of difference in associations between the endotheliopathy biomarkers and 30-day all-cause mortality in patients with or without septic shock or respiratory infection (Additional File 3–5: Figs. S1–S3). We found no difference in the associations between the endotheliopathy biomarkers and 30-day all-cause mortality among the sub-groups added post-hoc (Additional File 3–5: Figs. S1–S3).

Analyses of the biomarkers as categorical variables gave the same findings, with increasing rates of death for each increase in quartile for all three biomarkers (Fig. 5).

ROC-curve analysis showed that sTM was superior to both Syndecan-1 (AUC 0.66 vs. 0.59, *p* < 0.01) and CRP (AUC 0.66 vs. 0.51, *p* < 0.01) for predicting 30-day all-cause mortality. PECAM-1 was also superior to CRP in this regard (AUC 0.61 vs. 0.51, *p* = 0.03), but all other comparisons were non-significant (Additional File 8: Fig. S6). The optimal cut-point for predicting 30-day all-cause mortality determined with the Youden-index was 105.7 ng/ml for Syndecan-1, 10.1 ng/ml for sTM, and 13.2 ng/ml for PECAM-1.

### PaO_2_/FiO_2_-ratio

The worst PAF ratio on first day of ICU admission had a median value of 21.4 kPa (IQR 15.0–28.0).

For sTM (12% decrease in PAF-ratio with an increase from the 25th to the 75th percentile [*p* < 0.01]) and PECAM-1 (9% decrease in PAF-ratio with an increase from the 25th to the 75th percentile [*p* < 0.01]) higher levels were associated with lower PAF-ratio on the First Day of ICU-admission, whereas Syndecan-1 was not significantly associated with this outcome after adjustment (Additional File 9: Fig. S7 & Additional File 2: Tables S18–S20). On the first day of ICU admission, the highest OI was 5.8 (3.9–10.1) and the highest VR was 1.6 (1.2–2.0). Higher levels of sTM and PECAM-1 were associated with higher OI on the first day of ICU admission, and higher levels of sTM were associated with higher VR on the first day of ICU-admission (Additional File 2: Tables S21–S26).

To clarify the association between the three endotheliopathy biomarkers and the severity of respiratory failure at time of death in non-survivors, we modeled the PAF-ratio on the last measurement in patients dying during the first five ICU days. There was no evidence of association between any of the three biomarkers and the last measured PAF-ratio in neither the adjusted nor the unadjusted analyses (Additional File 10: Fig. S8 & Additional File 2: Tables S18–S20). Likewise, we found no associations with any of the endotheliopathy biomarkers or the last measured OI or VR in patients dying within the first five ICU days (Additional File 2: Tables S21–S26).

## Discussion

We tested the association of endotheliopathy—indicated by Syndecan-1, sTM, and PECAM-1 levels in plasma—with patient outcomes and disease severity in acute respiratory failure. We found that endotheliopathy biomarkers were independently associated with lower rates of liberation from MV, hypoxemia at ICU admission, higher rates of dying on MV, and higher 30-day all-cause mortality.

To our knowledge, this is the first study to test the impact of these three biomarkers together on patient outcomes in mechanically ventilated patients. Our findings show that some of the variation in outcomes observed in the population of patients with acute respiratory failure can be explained by the degree of endotheliopathy at ICU admission. This is potentially clinically relevant, as endotheliopathy may be a modifiable disease process and not just an unspecific marker of illness severity. A newly published randomized trial by some of the members of our author group tested a targeted treatment with a prostacyclin-2 analog, Iloprost, in critically ill COVID-19 patients with evidence of endotheliopathy [36]. The trial was underpowered but showed promising results with all point estimates in favor of the Iloprost-treatment. Two other randomized trials testing Iloprost in trauma and septic shock patients with evidence of endotheliopathy are currently ongoing and will provide more evidence if endotheliopathy is indeed a modifiable pathology or not [37, 38].

We found that sTM most consistently correlated with the outcomes studied. In existing literature, sTM is the most studied biomarker in acute respiratory failure. sTM was not associated with in-hospital mortality in a small observational study of patients with acute lung injury (*n* = 50) [30], nor with development of acute respiratory failure in a posthoc analysis of a large RCT of septic patients (*n* = 1103) [19]. In contrast, sTM correlated with: fewer ventilator-free days in an unadjusted analysis of a small RCT (*n* = 75) [26]; increased 60-day mortality in an adjusted analysis of an RCT including patients with ARDS (*n* = 449) [27]; ICU mortality in children with indirect causes of ARDS in a larger observational study after adjustment (*n* = 243) [28]; and 90-day mortality and oxygenation index in children with ARDS in a prospective cohort (*n* = 432) [29]. Our findings add to the existing evidence of sTM being an endotheliopathy biomarker that correlates well with outcomes of acute respiratory failure.

In previous studies, Syndecan-1 was associated with: the need for intubation after large-volume resuscitation in a prospective observational study (*n* = 175) [23]; the risk of developing respiratory failure in a prospective cohort of patients with sepsis from pneumonia (*n* = 44) [24]; and the risk for developing ARDS in a retrospective cohort of severely septic patients (*n* = 262) [25]. Syndecan-1 was not associated with the development of acute respiratory failure in the abovementioned posthoc analysis of an RCT on septic patients (*n* = 1103) [19]. To summarize, the Syndecan-1 has previously been tested as a marker for the development of acute respiratory failure, and the question of whether an association exists is equivocal. We found that Syndecan-1 showed the weakest associations with the outcomes studied, and in line with Johansen et al. [19] it could be argued that glycocalyx shedding in isolation is too early and non-specific a sign to properly differentiate between levels of severity in critically ill patients.

For PECAM-1, there are no clinical studies in humans to compare our results to. We did find that levels of PECAM-1 are increased in animal models of ventilator-associated lung injury [39, 40], implying a link between mechanical disruption of endothelial cell–cell junctions and PECAM-1 levels in blood. Our findings suggest PECAM-1 as a promising marker for clinical outcomes in acute respiratory failure.

Overall, while only patients with higher levels of sTM had lower global rates of liberation from mechanical ventilation, higher levels of all three biomarkers were associated with higher 30-day all-cause mortality. For sTM and PECAM-1, higher levels were associated with lower PAF-ratios on the first day of ICU admission but none of the three biomarkers showed association with the last measured PAF-ratio in patients dying within five days. To summarize, we found stronger associations with death and initial disease severity than with liberation from MV. This might be explained by the fact that even in patients with ARDS, the cause of death is rarely refractory respiratory failure or hypoxemia [41, 42]. Although we do not have data on the cause of death in our population, and keeping the limitations inherent to cause-of-death studies in mind, it is reasonable to suggest that our patients also died from multiple other causes than from respiratory failure directly, thereby at least partly explaining our findings.

Although our study has a relatively large sample size, published a statistical analysis plan before carrying out the analyses, and had complete follow-up, it is important to notice several limitations.

First, no screening log was kept during the study inclusion, so we cannot confirm the rate of inclusion or compare the characteristics of included versus non-included patients. The high SAPS 3 in the cohort suggests a tendency to include the ‘sicker’ patient when faced with limited time and resources. This, along with the single-center nature of the study, limits the external validity of our findings.

Second, we included patients receiving MV and did not apply a more rigorous definition of ARDS or acute lung injury. It is generally a strength to use pragmatical inclusion criteria, and the sub-group analyses showed very limited heterogeneity in associations pointing to that endotheliopathy is an important risk factor for worse outcomes in a diverse spectrum of patients with acute respiratory failure. However, the broad inclusion criteria also limit the extent to which our findings can be compared with previous studies applying more strict inclusion criteria.

Third, although liberation from MV is an objective and patient-important outcome, it is also affected by multiple other factors apart from the pathophysiology and illness severity of the patient such as patient and provider preferences, resource availability, and guidelines for provision of care. Therefore, we cannot establish a direct connection between liberation from MV and pulmonary pathophysiology, and the pathophysiological inferences from the present findings should be taken with caution.

Fourth, although our chosen biomarkers, especially Syndecan-1 and sTM, have been used as endotheliopathy markers in multiple prior studies, all our three biomarkers are found in different tissues apart from the endothelium. Syndecan-1 is expressed in multiple organs including the liver, digestive tract, and kidneys [13]; sTM is found in monocytes and epithelial cells [43, 44]; and PECAM-1 in leukocytes and thrombocytes [21, 22]. It is not possible to differentiate between different sources of the biomarkers found in plasma, and therefore we cannot exclude the possibility that a significant part of the measured biomarkers stem from other cell types than the endothelium.

Finally, it is worth mentioning that the time-varying analysis establishing the association between liberation of MV and PECAM-1 was performed posthoc. However, the analysis was decided upon after routine tests of important model assumptions and markedly improved the model fit to the data. We, therefore, consider the time-varying analysis to be part of a standard modeling procedure and find it prudent to place emphasis on the results despite the posthoc nature. It is also worth commenting that in the > 5 to 12-day period, higher levels of Syndecan-1 and PECAM-1 were associated with higher rates of liberation from MV. This seemingly paradoxical finding reflects that primarily in the groups with higher levels of Syndecan-1 and PECAM-1, were patients still on MV after 5 days. These patients were then liberated from MV in the > 5 to 12-day period. This indicates that a higher degree of endotheliopathy is associated with a prolonged time to recovery from acute respiratory failure which is in line with the rest of our findings.

## Conclusion

We found that higher plasma concentrations of endotheliopathy markers measured at ICU admission were independently associated with (1) lower rates of liberation from mechanical ventilation, (2) more pronounced hypoxemia on ICU admission, (3) higher rates of dying while still on mechanical ventilation and (4) increased all-cause 30-day mortality. Our findings point to endotheliopathy as an important factor in the development of acute respiratory failure.

## Supplementary Information

**Additional file 1.** Statistical Analysis Plan. Contains the preplanned Statistical Analysis Plan as published on our institutions website ahead of performing the final analyses.

**Additional file 2. Tables S1–S26**.

**Additional file 3. Fig. S1**: Associations of Syndecan-1 with liberation from mechanical ventilation, death on mechanical ventilation and 30-day all-cause mortality in various subgroups of patients with acute respiratory failure in the ICU. Hazard rates are from multivariable Cox-regression. All models were adjusted for age, gender, chronic obstructive pulmonary disease, heart failure, bilirubin, PaO2/FiO2-ratio, septic shock and respiratory infection.

**Additional file 4. Fig. S2**: Associations of Soluble Thrombomodulin (sTM) with liberation from mechanical ventilation, death on mechanical ventilation and 30-day all-cause mortality in various subgroups of patients with acute respiratory failure in the ICU. Hazard rates are from multivariable Cox-regression. All models were adjusted for age, gender, chronic obstructive pulmonary disease, heart failure, bilirubin, PaO2/FiO2-ratio, septic shock and respiratory infection.

**Additional file 5. Fig. S3**: Associations of Platelet Endothelial Cell Adhesion Molecule-1 (PECAM-1) with liberation from mechanical ventilation, death on mechanical ventilation and 30-day all-cause mortality in various subgroups of patients with acute respiratory failure in the ICU. Hazard rates are from multivariable Cox-regression. All models were adjusted for age, gender, chronic obstructive pulmonary disease, heart failure, bilirubin, PaO2/FiO2-ratio, septic shock and respiratory infection.

**Additional file 6. Fig. S4**: Reciever Operating Characteristics-curves for Syndecan-1, Soluble Thrombomodulin (sTM), Platelet Endothelial Cell Adhesion Molecule-1 (PECAM-1) and C-reactive Protein (CRP) for predicting the event of liberation from mechanical ventilation at 29 days from study inclusion in patients with acute respiratory failure in the ICU. Values are obtained from univariable competing risks Cox-regressions using the Score-function from the riskRegression R-package by Gerds et al.

**Additional file 7. Fig. S5**: Reciever Operating Characteristics-curves for Syndecan-1, Soluble Thrombomodulin (sTM), Platelet Endothelial Cell Adhesion Molecule-1 (PECAM-1) and C-reactive Protein (CRP) for predicting the event of death on mechanical ventilation at 29 days from study inclusion in patients with acute respiratory failure in the ICU. Values are obtained from univariable competing risks Cox-regressions using the Score-function from the riskRegression R-package by Gerds et al.

**Additional file 8. Fig. S6**: Reciever Operating Characteristics-curves for Syndecan-1, Soluble Thrombomodulin (sTM), Platelet Endothelial Cell Adhesion Molecule-1 (PECAM-1) and C-reactive Protein (CRP) for predicting 30-day all-cause mortality in patients with acute respiratory failure in the ICU. Values are obtained from univariable logistic regressions using the Score-function from the riskRegression R-package by Gerds et al.

**Additional file 9. Fig. S7**: Estimated PaO2/FiO2-ratio (PAF-ratio) in kPa on the first day of ICU-admssion with point estimates and 95% confidence intervals against the levels of Syndecan-1, soluble Thrombomodulin (sTM) and Platelet Endothelial Cell Adhesion Molecule-1 (PECAM-1) respectively. The estimates are adjusted for age, chronic obstructive pulmonary disease, shock (need for vasopressor & lactate > 2 at ICU-admission regardless of ethology) and respiratory infection.

**Additional file 10. Fig. S8** : Estimated last measured PaO2/FiO2-ratio (PAF-ratio) in kPa in patients dying within five days of ICU-admssion with point estimates and 95% confidence intervals against the levels of Syndecan-1, soluble Thrombomodulin (sTM) and Platelet Endothelial Cell Adhesion Molecule-1 (PECAM-1) respectively. The estimates are adjusted for age, chronic obstructive pulmonary disease, shock (need for vasopressor & lactate > 2 at ICU-admission regardless of ethology) and respiratory infection.

## Data Availability

The datasets used and/or analyzed during the current study are available from the corresponding author on reasonable request.

## Abbreviations

95% CI: 95% confidence interval
ARDS: Acute Respiratory Distress Syndrome
COPD: Chronic obstructive pulmonary disease
CRP: C-reactive protein
HR: Hazard ratio
ICU: Intensive care unit
IQR: Interquartile range
NIV: Non-invasive ventilation
MV: Mechanical ventilation
PAF-ratio: PaO_2_/FiO_2_-ratio
PECAM-1: Platelet Endothelial Cell Adhesion Molecule-1
RCT: Randomized controlled trial
ROC: Receiver Operating Characteristics
SAPS 3: Simplified Acute Physiology Score 3
SOFA: Sequential Organ Failure Assessment
sTM: Soluble thrombomodulin
