# Supplementary material for: Endotheliopathy is associated with slower liberation from mechanical ventilation: a cohort study
Source: Crit Care. 2022 Jan 30;26:33. doi: 10.1186/s13054-021-03877-y (PMC8801241; doi:10.1186/s13054-021-03877-y)
Supplement: Supplementary file 1 — Additional file 1. Statistical Analysis Plan. Contains the preplanned Statistical Analysis Plan as published on our institutions website ahead of performing the final analyses. [file 13054_2021_3877_MOESM1_ESM.docx]

# e-Appendix

Statistical Analysis plan for: ‘The effect of endothelial dysfunction on liberation from mechanical ventilation– a cohort study’

Authors: Martin Schønemann-Lund, Theis S. Itenov, Johan E. Larsson, Birgitte L. Madsen, Pär I. Johansson and Morten H. Bestle

# Introduction

### Background and Rationale

Acute respiratory failure is a common reason for admission to the Intensive Care Unit (ICU), and it is estimated that 13-20 million patients are mechanically ventilated worldwide annually^1^ with more than a third dying during the hospital admission, although studies suggest that the mortality have improved in recent years.^2–45^

Endothelial dysfunction has been proposed as a plausible link between critical illness and multiorgan failure^6^ and two Danish Randomized Controlled Trials (RCT) are currently testing the prostacyclin analogue Ilomedin as a targeted treatment against endotheliopathy in trauma and septic shock.

In experimental models, pathologic stimuli to the pulmonary endothelium results in the inflammation and noncardiogenic pulmonary oedema that are hallmarks of ARDS.^7–9^

In ARDS increased permeability of the intercellular connections between endothelial cells leads to fluid accumulating in the alveoli. A yet unpublished study found that Platelet Endothelial Cell Adhesion Molecule (PECAM-1) is the best marker for this interruption of the cell-cell junctions between the endothelial cells.

In clinical studies, soluble thrombomodulin (sTM) and Syndecan-1 are valid markers of damage to the profound endothelium and endothelial glycocalyx, respectively.^10^ In critically ill patients, both markers increase with more severe disease and predicts liver and renal failure.^10,11^ Increased sTM levels are associated to fewer ventilator free days,^12^ organ failure^13,14^ and mortality^14^ in patients with ARDS.

Syndecan-1 associates with acute respiratory failure in patients with pulmonary sepsis^15^ and severe sepsis after resuscitation with large volumes of fluids.^16^ Nevertheless, surprisingly both markers correlates poorly with hypoxaemia at ICU admission and need for mechanical ventilation.^10^

### Objectives

The broader aim of the study is to expand the knowledge on the connection between endothelial dysfunction and patient-important outcomes in the setting of critically ill patients with acute respiratory failure. If a connection between the duration of mechanical ventilation and endothelial dysfunction is found it could lead to better prognostication and identification of the sickest patients at ICU-admission. This could pave the way for clinical trials testing a targeted treatment against the endotheliopathy to shorten the time that mechanical ventilation is required.

The objective of the study is to estimate the impact of endothelial dysfunction as measured with sTM, Syndecan-1 and PECAM-1 on the time to liberation from mechanical ventilation in a general ICU population admitted with acute respiratory failure.

# Methods

### Study design

The Metabolomics study investigates the role of the endothelium in patient-important outcomes in a cohort of critically ill adults admitted to the ICU.

We present here an analysis of a subpopulation of the Metabolomics study.

The study is reported in accordance with the STROBE-recommendations^17^

### Sample size

As previous data on the levels of sTM, Syndecan-1 and PECAM-1 in a critically ill population of patients with acute respiratory failure are very scarce, formal power calculations were not possible to perform and the results should be considered exploratory.

### Outcome Assessment

Patients were followed for 30 days after inclusion for collection of clinical data from their ICU-stay including timing of liberation from mechanical ventilation and were subsequently followed for a year for the occurrence of death from any cause.

# Statistical Principles

A p-value below 0.05 is considered significant. 95% Confidence Intervals (95% CI) are presented along with point estimates where feasible.

Results from both the univariable and multivariable analysis are presented where relevant.

No correction for multiple comparisons is performed, as all study results will be regarded as exploratory.

# Trial Population

### Screening data

The ICU at Copenhagen University Hospital – North Zealand, Copenhagen, Denmark is a 12 bed, mixed surgical/medical ICU with around 900 admissions per year.

Between November 2016 and June 2019 all patients presenting to the ICU were screened for inclusion in the Metabolomics Study.

Unfortunately, no screening log was kept at the time.

### Eligibility

The inclusion criteria consisted of age > 18, acutely admitted to the ICU and a presumed stay > 24 h. The exclusion criteria were non-obtainable informed consent or active treatment deemed futile by the treating clinician.

Included patients had study blood samples drawn daily for the first 5 days of their ICU-stay or until death or discharge, whichever came first.

For the present study in the Metabolomics cohort, only patients that presented with acute respiratory failure are included. Acute respiratory failure is defined as patients receiving either Invasive (IV) or Non-Invasive Ventilation (NIV) on the first day of ICU-admission.

### Withdrawal

Reasons for withdrawal were if patient were included in error (did not fulfil inclusion criteria or did fulfil one or more exclusion criteria as well if patients were enrolled in the study at a previous ICU-stay) or if patient or relatives did not consent to participation in the study.

### Baseline Patient Characteristics

The characteristics collected at baseline include:

- Patient demographics (age and gender)
- Chronic comorbidities (a history of heart failure, hypertension, diabetes, stroke, prior MI etc.)
- Primary reason for ICU-admission (as per SAPS 3 categories)
- Category of ICU-admission (medical, surgical, acute, elective)
- Timeline parameters (time of inclusion, time of ICU-admission, time of hospital admission etc.)
- Infection status at admission
- ICU-admission source (operating theatre, ward, emergency department etc.)
- Physiology at baseline (PAF-ratio, blood pressure, pulse etc.)
- Therapy at baseline (noradrenaline, insulin etc.)
- Respiratory therapy at baseline

# Analysis

### Outcome Definitions

The primary outcome is time in days to liberation from mechanical ventilation. Liberation from mechanical ventilation is defined to occur on the second of two consecutive ICU-days without the need for either IV or NIV. Liberation from mechanical ventilation was also considered to occur on the day of ICU discharge if cessation of mechanical ventilation occurs on the same day as ICU-discharge in patients that are not readmitted to the ICU within two days. The competing risk of death is said to occur if patients died before or within 24 hours from liberation from mechanical ventilation. Patients are censored if they were moved to a different hospital while still receiving mechanical ventilation or at 30 days if still receiving mechanical ventilation.

Secondary outcomes are the risk of death from any cause at 30 days, PaO2/FiO2-ratio (PAF-ratio) on the first day of ICU-admission and last measured PAF-ratio in patients dying within 5 days from inclusion.

### Variables

The explanatory variables of interest are sTM, Syndecan-1 and PECAM-1. These are continuous and presented in ng/ml. They will also be introduced to the regression models as categorical variables divided into three groups: =< 25^th^ percentile, > 25^th^ and =< 75^th^ percentile, > 75^th^ percentile

To be controlled for in the multivariable analyses are the following known predictors of poor outcomes in the ICU: Gender, Age, A history of Chronic Inflammatory Pulmonary Disease (COPD), Septic Shock at ICU-admission, A history of Heart Failure, Worst PAF-ratio on the first day of ICU-admission, The presence of Respiratory Infection at ICU-admission, Acute Kidney Injury during the first day of ICU-admission and highest Bilirubin during first day of ICU-admission.

Septic shock is defined in accordance with the “Third international consensus definition for sepsis” as suspected or confirmed infection plus the need of vasoactive medication to maintain a mean arterial blood pressure of 65 and a blood lactate of 2 or more.^18^

COPD, Heart Failure and Acute Respiratory Infection are adjudicated by one author (MSL) based on information from the EMR without knowledge of the results of the primary outcome or of the values of sTM, Syndecan-1 or PECAM-1.

PAF-ratio is calculated with information from the EMR as the values of PaO2 and FiO2 giving the lowest ratio on a given day of ICU-admission.

Acute Kidney Injury is defined as stage 2 or more from the KDIGO guidelines and calculated based on the worst creatinine during the first day of ICU-admission.^19^ Baseline creatinine is defined as the median of all creatinine levels from 180 to 8 days prior to ICU-admission. In patients without a baseline creatinine, the creatinine levels is estimated with revised Lund-Malmö formula assuming a normal GFR of 70 ml/min/1,73m2.^20^

### Analysis Methods

The effect of sTM, Syndecan-1 and PECAM-1 on the primary outcome is analysed using cox regression and reported as cause-specific hazards using the method of Ozenne et al.^21^ A separate model is fitted for each of the explanatory variables of interest and each model is controlled for the abovementioned variables.

The effect of the markers of endothelial damage on the risk of death from any cause at 30 days is modelled with cox regression and controlled for the same variables as the analysis of the primary outcome.

We model the effects of continuous variables with restricted cubic splines with 2 knots^22^ if the introduction of splines significantly improves model fit as evaluated by the Wald Statistic.

The effect of the markers of endothelial damage on the PAF-ratio on the first day of ICU-admission and on last PAF-ratio in patients dying within 5 days of inclusion is modelled with linear regression and controlled for COPD, age, respiratory infection and shock (defined as need for vasopressor and a lactate >= 2).

All models will be examined for standard measures of model fit and assumptions and the included variables will be transformed as appropriately. We test for biological plausible interactions such as between levels of the biomarkers of endothelial damage and septic shock or respiratory infection. Interaction terms will be kept in the model if they are significant in the multivariable analysis based on the p-value for the effect of the interaction term.

### Missing Data

If less than 2,5% of an outcome or variable is missing, missing data is handled by case-wise deletion. If more than 2,5% is missing, multiple imputation will be performed using chained equations with predictive mean matching and logistic regression for numerical and categorical variables respectively.^23^ For the imputations all above mentioned variables and outcomes will be used to inform the imputations. 10 datasets will be imputed.

### Statistical programming software

All analyses are performed with R version 3.6.1 (The R Foundation for Statistical Computing).

### References

1. Adhikari NKJ, Fowler RA, Bhagwanjee S, Rubenfeld GD. Critical care and the global burden of critical illness in adults. *Lancet*. 2010;376(9749):1339-1346. doi:10.1016/S0140-6736(10)60446-1

2. Esteban A, Frutos-Vivar F, Muriel A, et al. Evolution of Mortality over Time in Patients Receiving Mechanical Ventilation. *Hosp Obrero*. 23(1). doi:10.1164/rccm.201212-2169OC

3. Erickson SE, Martin GS, Davis JL, Matthay MA, Eisner MD. Recent Trends in Acute Lung Injury Mortality: 1996-2005. doi:10.1097/CCM.0b013e31819fefdf

4. Zambon M, Vincent JL. Mortality rates for patients with acute lung injury/ARDS have decreased over time. *Chest*. 2008;133(5):1120-1127. doi:10.1378/chest.07-2134

5. Wunsch H, Linde-Zwirble WT, Angus DC, Hartman ME, Milbrandt EB, Kahn JM. The epidemiology of mechanical ventilation use in the United States. *Crit Care Med*. 2010;38(10):1947-1953. doi:10.1097/CCM.0b013e3181ef4460

6. Johansson PI, Stensballe J, Ostrowski SR. Shock induced endotheliopathy (SHINE) in acute critical illness - a unifying pathophysiologic mechanism. *Crit Care*. 2017;21(1):1-7. doi:10.1186/s13054-017-1605-5

7. Aird WC. Phenotypic heterogeneity of the endothelium: II. Representative vascular beds. *Circ Res*. 2007;100(2):174-190. doi:10.1161/01.RES.0000255690.03436.ae

8. Maniatis N, Orfanos S. The endothelium in acute respiratory distress syndrome. *Curr Opin Crit Care*. 2008:22-30. doi:10.1017/CBO9780511546198.129

9. Schmidt EP, Yang Y, Janssen WJ, et al. The pulmonary endothelial glycocalyx regulates neutrophil adhesion and lung injury during experimental sepsis. *Nat Med*. 2012;18(8):1217-1223. doi:10.1038/nm.2843

10. Johansen ME, Johansson PI, Ostrowski SR, et al. Profound Endothelial Damage Predicts Impending Organ Failure and Death in Sepsis. *Semin Thromb Hemost*. 2015;41:16-25. doi:10.1055/s-0034-1398377

11. Itenov TS, Jensen JU, Ostrowski SR, et al. Endothelial Damage Signals Refractory Acute Kidney Injury in Critically Ill Patients. *Shock*. 2017;47(6):696-701. doi:10.1097/SHK.0000000000000804

12. Agrawal A, Zhuo H, Brady S, et al. Pathogenetic and predictive value of biomarkers in patients with ALI and lower severity of illness: results from two clinical trials. *Am J Physiol Cell Mol Physiol*. 2012;303(8):L634-L639. doi:10.1152/ajplung.00195.2012

13. Orwoll BE, Spicer AC, Zinter MS, et al. Elevated soluble thrombomodulin is associated with organ failure and mortality in children with acute respiratory distress syndrome (ARDS): a prospective observational cohort study. *Crit Care*. 2015;19(1). doi:10.1186/s13054-015-1145-9

14. Sapru A, Calfee CS, Liu KD, et al. Plasma soluble thrombomodulin levels are associated with mortality in the acute respiratory distress syndrome. *Intensive Care Med*. 2015;41(3):470-478. doi:10.1007/s00134-015-3648-x

15. Smart L, Bosio E, Macdonald SPJ, et al. Glycocalyx biomarker syndecan-1 is a stronger predictor of respiratory failure in patients with sepsis due to pneumonia, compared to endocan. *J Crit Care*. 2018;47:93-98. doi:10.1016/j.jcrc.2018.06.015

16. Puskarich MA, Cornelius DC, Tharp J, Nandi U, Jones AE. Plasma syndecan-1 levels identify a cohort of patients with severe sepsis at high risk for intubation after large-volume intravenous fluid resuscitation. *J Crit Care*. 2016;36:125-129. doi:10.1016/j.jcrc.2016.06.027

17. von Elm E, Altman DG, Egger M, Pocock SJ, Gotzsche PC, Vandenbroucke JP. The Strengthening the Reporting of Observational Studies in Epidemiology (STROBE) statement: guidelines for reporting observational studies. *Lancet (London, England)*. 2007;370(9596):1453-1457. doi:10.1016/S0140-6736(07)61602-X

18. Singer, M. et al., Bellomo R, Bernard GR, et al. The third international consensus definitions for sepsis. *Jama*. 2016;315(8):801-810. doi:10.1001/jama.2016.0287.The

19. Kellum JA, Lameire N, Aspelin P, et al. Kidney disease: Improving global outcomes (KDIGO) acute kidney injury work group. KDIGO clinical practice guideline for acute kidney injury. *Kidney Int Suppl*. 2012;2(1):1-138. doi:10.1038/kisup.2012.1

20. Nyman U, Grubb A, Larsson A, et al. The revised Lund-Malmö GFR estimating equation outperforms MDRD and CKD-EPI across GFR, age and BMI intervals in a large Swedish population. *Clin Chem Lab Med*. 2014;52(6):815-824. doi:10.1515/cclm-2013-0741

21. Ozenne B, Sørensen AL, Scheike T, Torp-Pedersen C, Gerds TA. riskRegression: Predicting the risk of an event using cox regression models. *R J*. 2017;9(2):440-460. doi:10.32614/rj-2017-062

22. Gauthier J, Wu Q V., Gooley TA. Cubic splines to model relationships between continuous variables and outcomes: a guide for clinicians. *Bone Marrow Transplant*. 2020;55(4):675-680. doi:10.1038/s41409-019-0679-x

23. Buuren S van, Groothuis-Oudshoorn K. MICE: Multivariate Imputation by Chained Equations in R. *J Stat Softw*. 2011;45(3):1-67.
