# Supplementary material for: Endotheliopathy is associated with slower liberation from mechanical ventilation: a cohort study
Source: Crit Care. 2022 Jan 30;26:33. doi: 10.1186/s13054-021-03877-y (PMC8801241; doi:10.1186/s13054-021-03877-y)
Supplement: Supplementary file 2 — Additional file 2. Tables S1–S26. [file 13054_2021_3877_MOESM2_ESM.docx]

# Additional File 2: Tables

**Title:**

Endotheliopathy is associated with slower liberation from mechanical ventilation – a cohort study

**Authors:**

Martin Schønemann-Lund

Theis S. Itenov

Johan E. Larsson

Birgitte Lindegaard

Pär I. Johansson

Morten H. Bestle

**Table S1** Levels of soluble Thrombomodulin (sTM), Syndecan-1 and Platelet Endothelial Cell Adhesion Molecule-1 among patients with and without various chronic diseases (n = 459)

| *Disease state* | *No* | *Yes* | *p* |
| --- | --- | --- | --- |
| Chronic heart failure |  |  |  |
| sTM, ng/ml, median (IQR) | 10.02 (4.88 to 18.02) | 10.7 (6.89 to 17.13) | 0.5 |
| Syndecan-1, ng/ml, median (IQR) | 43.86 (20.95 to 118.49) | 70.53 (34.74 to 114.3) | 0.62 |
| PECAM-1, ng/ml, median (IQR) | 12.89 (11 to 14.51) | 13.8 (13.1 to 14.78) | **0.04** |
| Hypertension |  |  |  |
| sTM, ng/ml, median (IQR) | 8.89 (4.74 to 17.19) | 11.02 (5.23 to 18.31) | 0.14 |
| Syndecan-1, ng/ml, median (IQR) | 55.52 (25.64 to 153.19) | 41.69 (18 to 108.81) | **0.04** |
| PECAM-1, ng/ml, median (IQR) | 12.88 (10.85 to 14.6) | 13.19 (11.29 to 14.54) | 0.37 |
| COPD |  |  |  |
| sTM, ng/ml, median (IQR) | 11.75 (5.7 to 18.6) | 8.17 (3.66 to 14.66) | **< 0.01** |
| Syndecan-1, ng/ml, median (IQR) | 53.17 (24.75 to 127.14) | 36.4 (15.51 to 104.88) | 0.12 |
| PECAM-1, ng/ml, median (IQR) | 13.07 (11 to 14.52) | 13.23 (11.25 to 14.59) | 0.78 |
| Diabetes |  |  |  |
| sTM, ng/ml, median (IQR) | 8.7 (4.65 to 16.73) | 15.27 (7.69 to 20.98) | **< 0.01** |
| Syndecan-1, ng/ml, median (IQR) | 45.34 (23.33 to 116.35) | 47.47 (19.16 to 118.46) | 0.86 |
| PECAM-1, ng/ml, median (IQR) | 12.77 (10.8 to 14.49) | 13.61 (12.08 to 14.82) | **< 0.01** |
| Prior myocardial infarction |  |  |  |
| sTM, ng/ml, median (IQR) | 10.03 (5.04 to 18.02) | 10.42 (4.63 to 17.13) | 0.76 |
| Syndecan-1, ng/ml, median (IQR) | 49.46 (22.88 to 127.14) | 34.05 (17.18 to 96.38) | 0.12 |
| PECAM-1, ng/ml, median (IQR) | 13.04 (11 to 14.57) | 13.83 (11.59 to 14.54) | 0.38 |
| Active cancer within 6 months |  |  |  |
| sTM, ng/ml, median (IQR) | 10.06 (5.01 to 18.06) | 9.44 (5.1 to 16.19) | 0.56 |
| Syndecan-1, ng/ml, median (IQR) | 45.41 (21.02 to 127.14) | 39.96 (26.4 to 107.52) | 0.24 |
| PECAM-1, ng/ml, median (IQR) | 13.17 (11.19 to 14.62) | 11.99 (9.98 to 13.98) | 0.3 |
| Chronic kidney disease |  |  |  |
| sTM, ng/ml, median (IQR) | 8.6 (4.37 to 16.74) | 18.03 (13.11 to 21.2) | **< 0.01** |
| Syndecan-1, ng/ml, median (IQR) | 44.57 (20.59 to 114.39) | 61.09 (24.77 to 155.26) | 0.24 |
| PECAM-1, ng/ml, median (IQR) | 12.87 (10.86 to 14.52) | 13.67 (11.82 to 14.75) | **< 0.01** |

**Table S2** Treatments received by study patients during the first five days in the ICU

|  | | Day of ICU-stay | | | | |
| --- | --- | --- | --- | --- | --- | --- |
| *Treatment* | *Levels* | *1 (n = 459)* | *2 (n = 427)* | *3 (n = 348)* | *4 (n = 299)* | *5 (n = 252)* |
| Tidal volume, ml/kg | Median (IQR) | 8 (6.8 to 9.8) | 7.7 (6.5 to 9.5) | 7.8 (6.6 to 9.2) | 7.6 (6.5 to 9.1) | 7.4 (6.2 to 8.7) |
| PEEP, cm H2O | Median (IQR) | 7 (5 to 10) | 8 (5 to 10) | 8 (6 to 10) | 8 (6 to 12) | 8 (6 to 12) |
| Treatment with vasopressor* | n (%) | 307 (82) | 231 (66) | 172 (62) | 125 (53) | 100 (52) |
| Noradrenaline, mcg/kg/minute* | Median (IQR) | 0.17 (0.08 to 0.35) | 0.14 (0.08 to 0.33) | 0.12 (0.06 to 0.24) | 0.1 (0.04 to 0.18) | 0.09 (0.05 to 0.18) |
| Treatment with glucocorticoids* | n (%) | 78 (21) | 139 (40) | 117 (42) | 94 (40) | 58 (30) |
| Treatment with insulin* | n (%) | 63 (17) | 87 (25) | 70 (25) | 66 (28) | 53 (27) |
| Fluidbalance, ml† | Median (IQR) | 597 (1 to 2629) | 128 (-304 to 1017) | -1 (-724 to 635) | -190 (-976 to 152) | -395 (-1246 to 114) |
| Treatment with dialysis‡ | n (%) | 14 (4) | 24 (7) | 29 (10) | 29 (12) | 23 (12) |
| *Patients with data: Day 1 = 373, Day 2 = 351, Day 3 = 277, Day 4 = 236, Day 5 = 194 | | | | | | |
| †Patients with data: Day 1 = 370, Day 2 = 344, Day 3 = 282, Day 4 = 240, Day 5 = 204 | | | | | | |
| ‡Patients with data: Day 1 = 379, Day 2 = 349, Day 3 = 284, Day 4 = 241, Day 5 = 207 | | | | | | |

**Table S3** Association of Syndecan-1 as a continuous variable with liberation from mechanical ventilation and the competing risk of death on mechanical ventilation in mechanically ventilated patients in the intensive care unit – Cox-regression (n = 459)

|  | **Liberation from Mechanical Ventilation** | | | | **Death on Mechanical Ventilation** | | | |
| --- | --- | --- | --- | --- | --- | --- | --- | --- |
|  | *Univariable* | | *Multivariable* | | *Univariable* | | *Multivariable* | |
| *Predictor* | *Hazard Ratio (95% CI)* | *p* | *Hazard Ratio (95% CI)* | *p* | *Hazard Ratio (95% CI)* | *P* | *Hazard Ratio (95% CI)* | *p* |
| Syndecan-1, 25th vs. 75th percentile* | 0.84 (0.72-0.99) | **0.03** | 1.08 (0.91-1.3) | 0.38 | 1.47 (1.16-1.86) | **<0.01** | 1.47 (1.1-1.96) | **0.01** |
| Gender, male vs. Female | 0.81 (0.65-1.02) | 0.07 | 0.84 (0.66-1.06) | 0.14 | 1.02 (0.68-1.54) | 0.91 | 1.06 (0.7-1.61) | 0.79 |
| Age, years | 1.01 (1-1.02) | 0.19 | 1.01 (1-1.02) | 0.18 | 1.04 (1.02-1.06) | **<0.01** | 1.05 (1.02-1.07) | **<0.01** |
| COPD, yes vs. No | 1.77 (1.4-2.24) | **<0.01** | 1.54 (1.19-2) | **<0.01** | 1.39 (0.91-2.13) | 0.12 | 1.41 (0.9-2.22) | 0.13 |
| Chronic Heart Failure, yes vs. no | 0.8 (0.49-1.31) | 0.37 | 0.9 (0.55-1.49) | 0.69 | 2.21 (1.25-3.9) | **0.01** | 1.73 (0.95-3.15) | 0.07 |
| PaO2/FiO2-ratio, kPa† | 1.2 (1.08-1.33) | **<0.01** | 1.18 (1.06-1.31) | **<0.01** | 1.02 (0.89-1.16) | 0.81 | 0.97 (0.84-1.12) | 0.7 |
| Respiratory Infection, yes vs. no | 0.94 (0.75-1.18) | 0.62 | 0.98 (0.76-1.27) | 0.89 | 0.85 (0.57-1.26) | 0.41 | 0.81 (0.52-1.27) | 0.36 |
| Septic Shock, yes vs. no | 0.68 (0.5-0.93) | **0.02** | 0.81 (0.58-1.14) | 0.22 | 1.73 (1.13-2.67) | **0.01** | 1.34 (0.82-2.18) | 0.25 |
| Bilirubin, mmol/L† | 0.82 (0.71-0.95) | **0.01** | 0.85 (0.73-0.98) | **0.03** | 1.25 (0.86-1.81) | 0.24 | 1.16 (0.8-1.69) | 0.43 |
| KDIGO-score >= 2, yes vs. no | 0.77 (0.59-1.01) | 0.06 | 0.87 (0.66-1.16) | 0.36 | 1.63 (1.08-2.46) | **0.02** | 1.27 (0.8-2.02) | 0.31 |

*Syndecan-1 was analyzed as a continuous variable, and the HR associated with an increase from the 25^th^ to the 75^th^ percentile (21.0 – 118.4 ng/ml) is presented. † Modelled using a restricted cubic spline with 2 knots. COPD = Chronic Obstructive Pulmonary Disease, KDIGO-score =Kidney Disease, Improving Global Outcomes-score of acute kidney injury. 95 % CI = 95 % Confidence Interval

**Table S4** Association of soluble Thrombomodulin (sTM) as a continuous variable with liberation from mechanical ventilation and the competing risk of death on mechanical ventilation in mechanically ventilated patients in the intensive care unit – Cox-regression (n = 459)

|  | **Liberation from Mechanical Ventilation** | | | | **Death on Mechanical Ventilation** | | | |
| --- | --- | --- | --- | --- | --- | --- | --- | --- |
|  | *Univariable* | | *Multivariable* | | *Univariable* | | *Multivariable* | |
| *Predictor* | *Hazard Ratio (95% CI)* | *p* | *Hazard Ratio (95% CI)* | *p* | *Hazard Ratio (95% CI)* | *p* | *Hazard Ratio (95% CI)* | *p* |
| sTM, 25th vs. 75th percentile* | 0.57 (0.46-0.72) | **<0.01** | 0.71 (0.54-0.93) | **0.01** | 1.85 (1.28-2.7) | **<0.01** | 1.52 (0.95-2.45) | 0.08 |
| Gender, male vs. female | 0.81 (0.65-1.02) | 0.07 | 0.89 (0.7-1.13) | 0.33 | 1.02 (0.68-1.54) | 0.91 | 1.05 (0.69-1.6) | 0.81 |
| Age, years | 1.01 (1-1.02) | 0.19 | 1.01 (1-1.02) | 0.13 | 1.04 (1.02-1.06) | **<0.01** | 1.04 (1.02-1.07) | **<0.01** |
| COPD, yes vs. no | 1.77 (1.4-2.24) | **<0.01** | 1.47 (1.13-1.9) | **<0.01** | 1.39 (0.91-2.13) | 0.12 | 1.49 (0.94-2.34) | 0.09 |
| Chronic Heart Failure, yes vs. no | 0.8 (0.49-1.31) | 0.37 | 0.97 (0.59-1.6) | 0.9 | 2.21 (1.25-3.9) | **0.01** | 1.72 (0.95-3.12) | 0.08 |
| PaO2/FiO2-ratio, kPa † | 1.2 (1.08-1.33) | **<0.01** | 1.19 (1.07-1.32) | **<0.01** | 1.02 (0.89-1.16) | 0.81 | 0.97 (0.84-1.13) | 0.73 |
| Respiratory Infection, yes vs. no | 0.94 (0.75-1.18) | 0.62 | 0.96 (0.74-1.24) | 0.77 | 0.85 (0.57-1.26) | 0.41 | 0.84 (0.54-1.31) | 0.44 |
| Septic Shock, yes vs. no | 0.68 (0.5-0.93) | **0.02** | 0.83 (0.6-1.17) | 0.29 | 1.73 (1.13-2.67) | **0.01** | 1.39 (0.85-2.27) | 0.18 |
| Bilirubin, mmol/L † | 0.82 (0.71-0.95) | **0.01** | 0.86 (0.74-1) | 0.05 | 1.25 (0.86-1.81) | 0.24 | 1.19 (0.82-1.73) | 0.36 |
| KDIGO-score >= 2, yes vs. no | 0.77 (0.59-1.01) | 0.06 | 1.06 (0.77-1.45) | 0.73 | 1.63 (1.08-2.46) | **0.02** | 1.16 (0.71-1.89) | 0.56 |

* sTM was analyzed as a continuous variable, and the HR associated with an increase from the 25^th^ to the 75^th^ percentile (5.0 – 18.0 ng/ml) is presented. †Modelled using a restricted cubic spline with 2 knots. COPD = Chronic Obstructive Pulmonary Disease, KDIGO-score =Kidney Disease, Improving Global Outcomes-score of acute kidney injury. 95 % CI = 95 % Confidence Interval.

**Table S5** Association of Platelet Endothelial Cell Adhesion Molecule-1 (PECAM-1) as a continuous variable with liberation from mechanical ventilation and the competing risk of death on mechanical ventilation in mechanically ventilated patients in the intensive care unit – Cox-regression (n = 459)

|  | **Liberation from Mechanical Ventilation** | | | | **Death on Mechanical Ventilation** | | | |
| --- | --- | --- | --- | --- | --- | --- | --- | --- |
|  | *Univariable* | | *Multivariable* | | *Univariable* | | *Multivariable* | |
| *Predictor* | *Hazard Ratio (95% CI)* | *p* | *Hazard Ratio (95% CI)* | *p* | *Hazard Ratio (95% CI)* | *p* | *Hazard Ratio (95% CI)* | *p* |
| PECAM-1, 25th vs. 75th percentile* | 0.8 (0.69-0.93) | **<0.01** | 0.92 (0.78-1.08) | 0.31 | 1.57 (1.18-2.08) | **<0.01** | 1.49 (1.08-2.06) | **0.01** |
| Gender, male vs. female | 0.81 (0.65-1.02) | 0.07 | 0.84 (0.66-1.06) | 0.15 | 1.02 (0.68-1.54) | 0.91 | 1.09 (0.72-1.66) | 0.68 |
| Age, years | 1.01 (1-1.02) | 0.19 | 1.01 (1-1.02) | 0.21 | 1.04 (1.02-1.06) | **<0.01** | 1.05 (1.03-1.07) | **<0.01** |
| COPD, yes vs. no | 1.77 (1.4-2.24) | **<0.01** | 1.54 (1.19-2) | **<0.01** | 1.39 (0.91-2.13) | 0.12 | 1.37 (0.88-2.15) | 0.17 |
| Chronic Heart Failure, yes vs. no | 0.8 (0.49-1.31) | 0.37 | 0.93 (0.56-1.54) | 0.78 | 2.21 (1.25-3.9) | **0.01** | 1.66 (0.91-3.03) | 0.1 |
| PaO2/FiO2-ratio, kPa† | 1.2 (1.08-1.33) | **<0.01** | 1.18 (1.06-1.31) | **<0.01** | 1.02 (0.89-1.16) | 0.81 | 0.98 (0.85-1.13) | 0.78 |
| Respiratory Infection, yes vs. no | 0.94 (0.75-1.18) | 0.62 | 0.98 (0.76-1.27) | 0.88 | 0.85 (0.57-1.26) | 0.41 | 0.83 (0.53-1.3) | 0.42 |
| Septic Shock, yes vs. no | 0.68 (0.5-0.93) | **0.02** | 0.81 (0.58-1.13) | 0.22 | 1.73 (1.13-2.67) | **0.01** | 1.43 (0.88-2.32) | 0.15 |
| Bilirubin, mmol/L† | 0.82 (0.71-0.95) | **0.01** | 0.85 (0.74-0.99) | **0.04** | 1.25 (0.86-1.81) | 0.24 | 1.18 (0.81-1.71) | 0.39 |
| KDIGO-score >= 2, yes vs. no | 0.77 (0.59-1.01) | 0.06 | 0.91 (0.68-1.21) | 0.52 | 1.63 (1.08-2.46) | **0.02** | 1.3 (0.83-2.04) | 0.26 |

*PECAM-1 was analyzed as a continuous variable, and the HR associated with an increase from the 25^th^ to the 75^th^ percentile (11.1 – 14.6 ng/ml) is presented. †Modelled using a restricted cubic spline with 2 knots. COPD = Chronic Obstructive Pulmonary Disease, KDIGO-score =Kidney Disease, Improving Global Outcomes-score of acute kidney injury. 95 % CI = 95 % Confidence Interval.

**Table S6** Association of Syndecan-1 as a continuous variable with liberation from mechanical ventilation and the competing risk of death on mechanical ventilation in mechanically ventilated patients in the intensive care unit – Cox-regression controlled for oxygenation index (n = 459)

|  | **Liberation from Mechanical Ventilation** | | | | **Death on Mechanical Ventilation** | | | |
| --- | --- | --- | --- | --- | --- | --- | --- | --- |
|  | *Univariable* | | *Multivariable* | | *Univariable* | | *Multivariable* | |
| *Predictor* | *Hazard Ratio (95% CI)* | *p* | *Hazard Ratio (95% CI)* | *p* | *Hazard Ratio (95% CI)* | *p* | *Hazard Ratio (95% CI)* | *p* |
| Syndecan-1, 25th vs. 75th percentile* | 0.84 (0.72-0.99) | **0.03** | 1.08 (0.9-1.29) | 0.4 | 1.47 (1.16-1.86) | **<0.01** | 1.47 (1.11-1.96) | **0.01** |
| Sex, male vs. female | 0.81 (0.65-1.02) | 0.07 | 0.84 (0.66-1.07) | 0.17 | 1.02 (0.68-1.54) | 0.91 | 1.06 (0.7-1.61) | 0.77 |
| Age, years | 1.01 (1-1.02) | 0.19 | 1 (0.99-1.01) | 0.88 | 1.04 (1.02-1.06) | **<0.01** | 1.05 (1.02-1.07) | **<0.01** |
| COPD, yes vs. no | 1.77 (1.4-2.24) | **<0.01** | 1.45 (1.13-1.87) | **<0.01** | 1.39 (0.91-2.13) | 0.12 | 1.34 (0.86-2.07) | 0.2 |
| Chronic heart failure, yes vs. no | 0.8 (0.49-1.31) | 0.37 | 0.88 (0.53-1.46) | 0.62 | 2.21 (1.25-3.9) | **0.01** | 1.68 (0.93-3.06) | 0.09 |
| Oxygenation index | 0.91 (0.89-0.94) | **<0.01** | 0.91 (0.89-0.94) | **<0.01** | 0.99 (0.96-1.02) | 0.41 | 1 (0.97-1.03) | 0.96 |
| Respiratory infection, yes vs. no | 0.94 (0.75-1.18) | 0.62 | 1.01 (0.79-1.3) | 0.91 | 0.85 (0.57-1.26) | 0.41 | 0.82 (0.53-1.27) | 0.37 |
| Septic shock, yes vs. no | 0.68 (0.5-0.93) | **0.02** | 0.86 (0.61-1.21) | 0.39 | 1.73 (1.13-2.67) | **0.01** | 1.35 (0.83-2.18) | 0.22 |
| Bilirubin, mmol/L† | 0.82 (0.71-0.95) | **0.01** | 0.86 (0.74-1.01) | 0.06 | 1.25 (0.86-1.81) | 0.24 | 1.19 (0.81-1.74) | 0.37 |
| KDIGO-score >= 2, yes vs. no | 0.77 (0.59-1.01) | 0.06 | 0.86 (0.65-1.15) | 0.31 | 1.63 (1.08-2.46) | **0.02** | 1.25 (0.79-1.98) | 0.33 |

*Syndecan-1 was analyzed as a continuous variable, and the HR associated with an increase from the 25^th^ to the 75^th^ percentile (21.0 – 118.4 ng/ml) is presented. † Modelled using a restricted cubic spline with 2 knots. COPD = Chronic Obstructive Pulmonary Disease, KDIGO-score =Kidney Disease, Improving Global Outcomes-score of acute kidney injury. 95 % CI = 95 % Confidence Interval

**Table S7** Association of soluble Thrombomodulin (sTM) as a continuous variable with liberation from mechanical ventilation and the competing risk of death on mechanical ventilation in mechanically ventilated patients in the intensive care unit – Cox-regression controlled for oxygenation index (n = 459)

|  | **Liberation from Mechanical Ventilation** | | | | **Death on Mechanical Ventilation** | | | |
| --- | --- | --- | --- | --- | --- | --- | --- | --- |
|  | *Univariable* | | *Multivariable* | | *Univariable* | | *Multivariable* | |
| *Predictor* | *Hazard Ratio (95% CI)* | *p* | *Hazard Ratio (95% CI)* | *p* | *Hazard Ratio (95% CI)* | *p* | *Hazard Ratio (95% CI)* | *p* |
| sTM, 25th vs. 75th percentile* | 0.57 (0.46-0.72) | **<0.01** | 0.76 (0.58-1) | 0.05 | 1.85 (1.28-2.7) | **<0.01** | 1.5 (0.93-2.41) | 0.09 |
| Sex, male vs. female | 0.81 (0.65-1.02) | 0.07 | 0.88 (0.69-1.12) | 0.3 | 1.02 (0.68-1.54) | 0.91 | 1.06 (0.7-1.61) | 0.79 |
| Age, years | 1.01 (1-1.02) | 0.19 | 1 (0.99-1.01) | 0.77 | 1.04 (1.02-1.06) | **<0.01** | 1.04 (1.02-1.06) | **<0.01** |
| COPD, yes vs. no | 1.77 (1.4-2.24) | **<0.01** | 1.41 (1.09-1.82) | **0.01** | 1.39 (0.91-2.13) | 0.12 | 1.38 (0.89-2.14) | 0.15 |
| Chronic Heart Failure, yes vs. no | 0.8 (0.49-1.31) | 0.37 | 0.94 (0.57-1.56) | 0.81 | 2.21 (1.25-3.9) | **0.01** | 1.68 (0.93-3.03) | 0.09 |
| Oxygenation index | 0.91 (0.89-0.94) | **<0.01** | 0.92 (0.89-0.94) | **<0.01** | 0.99 (0.96-1.02) | 0.41 | 1 (0.97-1.03) | 0.81 |
| Respiratory Infection, yes vs. no | 0.94 (0.75-1.18) | 0.62 | 1 (0.78-1.28) | 0.99 | 0.85 (0.57-1.26) | 0.41 | 0.86 (0.55-1.33) | 0.49 |
| Septic Shock, yes vs. no | 0.68 (0.5-0.93) | **0.02** | 0.89 (0.64-1.25) | 0.5 | 1.73 (1.13-2.67) | **0.01** | 1.43 (0.88-2.3) | 0.15 |
| Bilirubin, mmol/L† | 0.82 (0.71-0.95) | **0.01** | 0.87 (0.75-1.02) | 0.08 | 1.25 (0.86-1.81) | 0.24 | 1.21 (0.83-1.77) | 0.31 |
| KDIGO-score >= 2, yes vs. no | 0.77 (0.59-1.01) | 0.06 | 1.01 (0.74-1.4) | 0.93 | 1.63 (1.08-2.46) | **0.02** | 1.14 (0.7-1.86) | 0.6 |

* sTM was analyzed as a continuous variable, and the HR associated with an increase from the 25^th^ to the 75^th^ percentile (5.0 – 18.0 ng/ml) is presented. †Modelled using a restricted cubic spline with 2 knots. COPD = Chronic Obstructive Pulmonary Disease, KDIGO-score =Kidney Disease, Improving Global Outcomes-score of acute kidney injury. 95 % CI = 95 % Confidence Interval.

**Table S8** Association of Platelet Endothelial Cell Adhesion Molecule-1 (PECAM-1) as a continuous variable with liberation from mechanical ventilation and the competing risk of death on mechanical ventilation in mechanically ventilated patients in the intensive care unit – Cox-regression controlled for oxygenation index (n = 459)

|  | **Liberation from Mechanical Ventilation** | | | | **Death on Mechanical Ventilation** | | | |
| --- | --- | --- | --- | --- | --- | --- | --- | --- |
|  | *Univariable* | | *Multivariable* | | *Univariable* | | *Multivariable* | |
| *Predictor* | *Hazard Ratio (95% CI)* | *p* | *Hazard Ratio (95% CI)* | *p* | *Hazard Ratio (95% CI)* | *p* | *Hazard Ratio (95% CI)* | *p* |
| PECAM-1, 25th vs. 75th percentile* | 0.8 (0.69-0.93) | **<0.01** | 0.89 (0.75-1.04) | 0.14 | 1.57 (1.18-2.08) | **<0.01** | 1.48 (1.08-2.02) | **0.02** |
| Sex, male vs. female | 0.81 (0.65-1.02) | 0.07 | 0.84 (0.66-1.07) | 0.16 | 1.02 (0.68-1.54) | 0.91 | 1.1 (0.72-1.67) | 0.65 |
| Age, years | 1.01 (1-1.02) | 0.19 | 1 (0.99-1.01) | 0.94 | 1.04 (1.02-1.06) | **<0.01** | 1.05 (1.02-1.07) | **<0.01** |
| COPD, yes vs. no | 1.77 (1.4-2.24) | **<0.01** | 1.47 (1.14-1.9) | **<0.01** | 1.39 (0.91-2.13) | 0.12 | 1.28 (0.83-1.98) | 0.27 |
| Chronic Heart Failure, yes vs. no | 0.8 (0.49-1.31) | 0.37 | 0.92 (0.55-1.52) | 0.73 | 2.21 (1.25-3.9) | **0.01** | 1.61 (0.89-2.91) | 0.12 |
| Oxygenation index | 0.91 (0.89-0.94) | **<0.01** | 0.92 (0.89-0.94) | **<0.01** | 0.99 (0.96-1.02) | 0.41 | 1 (0.97-1.03) | 0.82 |
| Respiratory Infection, yes vs. no | 0.94 (0.75-1.18) | 0.62 | 1.01 (0.79-1.3) | 0.92 | 0.85 (0.57-1.26) | 0.41 | 0.84 (0.54-1.29) | 0.42 |
| Septic Shock, yes vs. no | 0.68 (0.5-0.93) | **0.02** | 0.86 (0.61-1.21) | 0.38 | 1.73 (1.13-2.67) | **0.01** | 1.46 (0.91-2.34) | 0.12 |
| Bilirubin, mmol/L† | 0.82 (0.71-0.95) | **0.01** | 0.87 (0.75-1.01) | 0.07 | 1.25 (0.86-1.81) | 0.24 | 1.2 (0.82-1.75) | 0.34 |
| KDIGO-score >= 2, yes vs. no | 0.77 (0.59-1.01) | 0.06 | 0.91 (0.68-1.22) | 0.54 | 1.63 (1.08-2.46) | **0.02** | 1.27 (0.81-1.98) | 0.3 |

*PECAM-1 was analyzed as a continuous variable, and the HR associated with an increase from the 25^th^ to the 75^th^ percentile (11.1 – 14.6 ng/ml) is presented. †Modelled using a restricted cubic spline with 2 knots. COPD = Chronic Obstructive Pulmonary Disease, KDIGO-score =Kidney Disease, Improving Global Outcomes-score of acute kidney injury. 95 % CI = 95 % Confidence Interval.

**Table S9** Association of Syndecan-1 as a continuous variable with liberation from mechanical ventilation and the competing risk of death on mechanical ventilation in mechanically ventilated patients in the intensive care unit – Cox-regression controlled for ventilatory ratio (n = 459)

|  | **Liberation from Mechanical Ventilation** | | | | **Death on Mechanical Ventilation** | | | |
| --- | --- | --- | --- | --- | --- | --- | --- | --- |
|  | *Univariable* | | *Multivariable* | | *Univariable* | | *Multivariable* | |
| *Predictor* | *Hazard Ratio (95% CI)* | *p* | *Hazard Ratio (95% CI)* | *p* | *Hazard Ratio (95% CI)* | *p* | *Hazard Ratio (95% CI)* | *p* |
| Syndecan-1, 25th vs. 75th percentile* | 0.84 (0.72-0.99) | **0.03** | 1.08 (0.9-1.3) | 0.39 | 1.47 (1.16-1.86) | **<0.01** | 1.47 (1.1-1.97) | **0.01** |
| Sex, male vs. female | 0.81 (0.65-1.02) | 0.07 | 0.81 (0.63-1.04) | 0.1 | 1.02 (0.68-1.54) | 0.91 | 1.09 (0.71-1.67) | 0.71 |
| Age, years | 1.01 (1-1.02) | 0.19 | 1 (0.99-1.01) | 0.75 | 1.04 (1.02-1.06) | **<0.01** | 1.05 (1.02-1.07) | **<0.01** |
| COPD, yes vs. no | 1.77 (1.4-2.24) | **<0.01** | 1.8 (1.38-2.34) | **<0.01** | 1.39 (0.91-2.13) | 0.12 | 1.3 (0.83-2.05) | 0.26 |
| Chronic heart failure, yes vs. no | 0.8 (0.49-1.31) | 0.37 | 0.87 (0.51-1.47) | 0.6 | 2.21 (1.25-3.9) | **0.01** | 1.66 (0.9-3.06) | 0.11 |
| Ventilatory ratio† | 0.49 (0.17-1.38) | 0.18 | 0.49 (0.15-1.56) | 0.23 | 0.58 (0.06-5.34) | 0.63 | 0.73 (0.07-8.02) | 0.8 |
| Respiratory infection, yes vs. no | 0.94 (0.75-1.18) | 0.62 | 0.87 (0.67-1.12) | 0.28 | 0.85 (0.57-1.26) | 0.41 | 0.79 (0.5-1.25) | 0.32 |
| Septic shock, yes vs. no | 0.68 (0.5-0.93) | **0.02** | 0.82 (0.59-1.16) | 0.27 | 1.73 (1.13-2.67) | **0.01** | 1.37 (0.83-2.26) | 0.22 |
| Bilirubin, mmol/L† | 0.82 (0.71-0.95) | **0.01** | 0.81 (0.7-0.95) | **0.01** | 1.25 (0.86-1.81) | 0.24 | 1.2 (0.81-1.76) | 0.36 |
| KDIGO-score >= 2, yes vs. no | 0.77 (0.59-1.01) | 0.06 | 0.87 (0.65-1.17) | 0.37 | 1.63 (1.08-2.46) | **0.02** | 1.22 (0.77-1.96) | 0.4 |

*Syndecan-1 was analyzed as a continuous variable, and the HR associated with an increase from the 25^th^ to the 75^th^ percentile (21.0 – 118.4 ng/ml) is presented. † Modelled using a restricted cubic spline with 2 knots. COPD = Chronic Obstructive Pulmonary Disease, KDIGO-score =Kidney Disease, Improving Global Outcomes-score of acute kidney injury. 95 % CI = 95 % Confidence Interval

**Table S10** Association of soluble Thrombomodulin (sTM) as a continuous variable with liberation from mechanical ventilation and the competing risk of death on mechanical ventilation in mechanically ventilated patients in the intensive care unit – Cox-regression controlled for ventilatory ratio (n = 459)

|  | **Liberation from Mechanical Ventilation** | | | | **Death on Mechanical Ventilation** | | | |
| --- | --- | --- | --- | --- | --- | --- | --- | --- |
|  | *Univariable* | | *Multivariable* | | *Univariable* | | *Multivariable* | |
| *Predictor* | *Hazard Ratio (95% CI)* | *p* | *Hazard Ratio (95% CI)* | *p* | *Hazard Ratio (95% CI)* | *p* | *Hazard Ratio (95% CI)* | *p* |
| sTM, 25th vs. 75th percentile* | 0.57 (0.46-0.72) | **<0.01** | 0.73 (0.55-0.98) | **0.04** | 1.85 (1.28-2.7) | **<0.01** | 1.48 (0.9-2.42) | 0.12 |
| Sex, male vs. female | 0.81 (0.65-1.02) | 0.07 | 0.85 (0.66-1.1) | 0.21 | 1.02 (0.68-1.54) | 0.91 | 1.08 (0.7-1.66) | 0.74 |
| Age, years | 1.01 (1-1.02) | 0.19 | 1 (0.99-1.01) | 0.56 | 1.04 (1.02-1.06) | **<0.01** | 1.04 (1.02-1.07) | **<0.01** |
| COPD, yes vs. no | 1.77 (1.4-2.24) | **<0.01** | 1.69 (1.3-2.21) | **<0.01** | 1.39 (0.91-2.13) | 0.12 | 1.36 (0.86-2.16) | 0.19 |
| Chronic Heart Failure, yes vs. no | 0.8 (0.49-1.31) | 0.37 | 0.92 (0.54-1.56) | 0.75 | 2.21 (1.25-3.9) | **0.01** | 1.64 (0.89-3.02) | 0.11 |
| Ventilatory ratio† | 0.49 (0.17-1.38) | 0.18 | 0.51 (0.17-1.53) | 0.23 | 0.58 (0.06-5.34) | 0.63 | 0.67 (0.06-7.22) | 0.74 |
| Respiratory Infection, yes vs. no | 0.94 (0.75-1.18) | 0.62 | 0.85 (0.66-1.1) | 0.22 | 0.85 (0.57-1.26) | 0.41 | 0.83 (0.53-1.3) | 0.41 |
| Septic Shock, yes vs. no | 0.68 (0.5-0.93) | **0.02** | 0.86 (0.61-1.21) | 0.38 | 1.73 (1.13-2.67) | **0.01** | 1.45 (0.88-2.38) | 0.15 |
| Bilirubin, mmol/L† | 0.82 (0.71-0.95) | **0.01** | 0.83 (0.71-0.96) | **0.01** | 1.25 (0.86-1.81) | 0.24 | 1.22 (0.83-1.78) | 0.31 |
| KDIGO-score >= 2, yes vs. no | 0.77 (0.59-1.01) | 0.06 | 1.04 (0.75-1.44) | 0.83 | 1.63 (1.08-2.46) | **0.02** | 1.12 (0.68-1.85) | 0.65 |

* sTM was analyzed as a continuous variable, and the HR associated with an increase from the 25^th^ to the 75^th^ percentile (5.0 – 18.0 ng/ml) is presented. †Modelled using a restricted cubic spline with 2 knots. COPD = Chronic Obstructive Pulmonary Disease, KDIGO-score =Kidney Disease, Improving Global Outcomes-score of acute kidney injury. 95 % CI = 95 % Confidence Interval.

**Table S11** Association of Platelet Endothelial Cell Adhesion Molecule-1 (PECAM-1) as a continuous variable with liberation from mechanical ventilation and the competing risk of death on mechanical ventilation in mechanically ventilated patients in the intensive care unit – Cox-regression controlled for ventilatory ratio (n = 459)

|  | **Liberation from Mechanical Ventilation** | | | | **Death on Mechanical Ventilation** | | | |
| --- | --- | --- | --- | --- | --- | --- | --- | --- |
|  | *Univariable* | | *Multivariable* | | *Univariable* | | *Multivariable* | |
| *Predictor* | *Hazard Ratio (95% CI)* | *p* | *Hazard Ratio (95% CI)* | *p* | *Hazard Ratio (95% CI)* | *p* | *Hazard Ratio (95% CI)* | *p* |
| PECAM-1, 25th vs. 75th percentile* | 0.8 (0.69-0.93) | **<0.01** | 0.89 (0.75-1.04) | 0.13 | 1.57 (1.18-2.08) | **<0.01** | 1.48 (1.08-2.02) | **0.02** |
| Sex, male vs. female | 0.81 (0.65-1.02) | 0.07 | 0.81 (0.63-1.04) | 0.1 | 1.02 (0.68-1.54) | 0.91 | 1.13 (0.73-1.73) | 0.59 |
| Age, years | 1.01 (1-1.02) | 0.19 | 1 (0.99-1.01) | 0.77 | 1.04 (1.02-1.06) | **<0.01** | 1.05 (1.02-1.07) | **<0.01** |
| COPD, yes vs. no | 1.77 (1.4-2.24) | **<0.01** | 1.8 (1.39-2.34) | **<0.01** | 1.39 (0.91-2.13) | 0.12 | 1.25 (0.79-1.97) | 0.33 |
| Chronic Heart Failure, yes vs. no | 0.8 (0.49-1.31) | 0.37 | 0.9 (0.53-1.53) | 0.7 | 2.21 (1.25-3.9) | **0.01** | 1.58 (0.85-2.91) | 0.15 |
| Ventilatory ratio† | 0.49 (0.17-1.38) | 0.18 | 0.49 (0.15-1.59) | 0.23 | 0.58 (0.06-5.34) | 0.63 | 0.71 (0.06-7.9) | 0.78 |
| Respiratory Infection, yes vs. no | 0.94 (0.75-1.18) | 0.62 | 0.86 (0.67-1.11) | 0.24 | 0.85 (0.57-1.26) | 0.41 | 0.8 (0.51-1.26) | 0.34 |
| Septic Shock, yes vs. no | 0.68 (0.5-0.93) | **0.02** | 0.83 (0.59-1.16) | 0.27 | 1.73 (1.13-2.67) | **0.01** | 1.47 (0.9-2.41) | 0.12 |
| Bilirubin, mmol/L† | 0.82 (0.71-0.95) | **0.01** | 0.82 (0.71-0.95) | **0.01** | 1.25 (0.86-1.81) | 0.24 | 1.21 (0.83-1.77) | 0.33 |
| KDIGO-score >= 2, yes vs. no | 0.77 (0.59-1.01) | 0.06 | 0.92 (0.69-1.24) | 0.61 | 1.63 (1.08-2.46) | **0.02** | 1.23 (0.77-1.95) | 0.38 |

*PECAM-1 was analyzed as a continuous variable, and the HR associated with an increase from the 25^th^ to the 75^th^ percentile (11.1 – 14.6 ng/ml) is presented. †Modelled using a restricted cubic spline with 2 knots. COPD = Chronic Obstructive Pulmonary Disease, KDIGO-score =Kidney Disease, Improving Global Outcomes-score of acute kidney injury. 95 % CI = 95 % Confidence Interval.

**Table S12** Association of Syndecan-1 as a continuous variable with liberation from mechanical ventilation and the competing risk of death on mechanical ventilation in mechanically ventilated patients in the intensive care unit – Cox-regression controlled for Simplified Acute Physiology Score 3 (SAPS 3) (n = 459)

|  | **Liberation from Mechanical Ventilation** | | | | **Death on Mechanical Ventilation** | | | |
| --- | --- | --- | --- | --- | --- | --- | --- | --- |
|  | *Univariable* | | *Multivariable* | | *Univariable* | | *Multivariable* | |
| *Predictor* | *Hazard Ratio (95% CI)* | *p* | *Hazard Ratio (95% CI)* | *p* | *Hazard Ratio (95% CI)* | *p* | *Hazard Ratio (95% CI)* | *p* |
| Syndecan-1, 25th vs. 75th percentile* | 0.84 (0.72-0.99) | **0.03** | 0.88 (0.75-1.03) | 0.12 | 1.47 (1.16-1.86) | **<0.01** | 1.31 (1.02-1.67) | **0.04** |
| SAPS 3 | 0.99 (0.98-0.99) | **<0.01** | 0.99 (0.98-1) | **<0.01** | 1.03 (1.02-1.05) | **<0.01** | 1.03 (1.02-1.04) | **<0.01** |

*Syndecan-1 was analyzed as a continuous variable, and the HR associated with an increase from the 25^th^ to the 75^th^ percentile (21.0 – 118.4 ng/ml) is presented. 95 % CI = 95 % Confidence Interval

**Table S13** Association of soluble Thrombomodulin (sTM) as a continuous variable with liberation from mechanical ventilation and the competing risk of death on mechanical ventilation in mechanically ventilated patients in the intensive care unit – Cox-regression controlled for Simplified Acute Physiology Score 3 (SAPS 3) (n = 459)

|  | **Liberation from Mechanical Ventilation** | | | | **Death on Mechanical Ventilation** | | | |
| --- | --- | --- | --- | --- | --- | --- | --- | --- |
|  | *Univariable* | | *Multivariable* | | *Univariable* | | *Multivariable* | |
| *Predictor* | *Hazard Ratio (95% CI)* | *p* | *Hazard Ratio (95% CI)* | *p* | *Hazard Ratio (95% CI)* | *p* | *Hazard Ratio (95% CI)* | *p* |
| sTM, 25th vs. 75th percentile* | 0.57 (0.46-0.72) | **<0.01** | 0.61 (0.49-0.77) | **<0.01** | 1.85 (1.28-2.7) | **<0.01** | 1.55 (1.05-2.28) | **0.03** |
| SAPS 3 | 0.99 (0.98-0.99) | **<0.01** | 0.99 (0.98-1) | **0.03** | 1.03 (1.02-1.05) | **<0.01** | 1.03 (1.02-1.05) | **<0.01** |

* sTM was analyzed as a continuous variable, and the HR associated with an increase from the 25^th^ to the 75^th^ percentile (5.0 – 18.0 ng/ml) is presented. 95 % CI = 95 % Confidence Interval.

**Table S14** Association of Platelet Endothelial Cell Adhesion Molecule-1 (PECAM-1) as a continuous variable with liberation from mechanical ventilation and the competing risk of death on mechanical ventilation in mechanically ventilated patients in the intensive care unit – Cox-regression controlled for Simplified Acute Physiology Score 3 (SAPS 3) (n = 459)

|  | **Liberation from Mechanical Ventilation** | | | | **Death on Mechanical Ventilation** | | | |
| --- | --- | --- | --- | --- | --- | --- | --- | --- |
|  | *Univariable* | | *Multivariable* | | *Univariable* | | *Multivariable* | |
| *Predictor* | *Hazard Ratio (95% CI)* | *p* | *Hazard Ratio (95% CI)* | *p* | *Hazard Ratio (95% CI)* | *p* | *Hazard Ratio (95% CI)* | *p* |
| PECAM-1, 25th vs. 75th percentile* | 0.8 (0.69-0.93) | **<0.01** | 0.82 (0.7-0.95) | **0.01** | 1.57 (1.18-2.08) | **<0.01** | 1.45 (1.08-1.94) | **0.01** |
| SAPS 3 | 0.99 (0.98-0.99) | **<0.01** | 0.99 (0.98-1) | **<0.01** | 1.03 (1.02-1.05) | **<0.01** | 1.03 (1.02-1.05) | **<0.01** |

*PECAM-1 was analyzed as a continuous variable, and the HR associated with an increase from the 25^th^ to the 75^th^ percentile (11.1 – 14.6 ng/ml) is presented.

**Table S15** Association of Syndecan-1 as a continuous variable with 30-day all-cause mortality in mechanically ventilated patients in the intensive care unit – Cox-regression (n = 459)

|  | **30-day all-cause mortality** | | | |
| --- | --- | --- | --- | --- |
|  | *Univariable* | | *Multivariable* | |
| *Predictor* | *Hazard Ratio (95% CI)* | *p* | *Hazard Ratio (95% CI)* | *p* |
| Syndecan-1, 25th vs. 75th percentile* | 1.44 (1.19-1.73) | **<0.01** | 1.36 (1.1-1.7) | **0.01** |
| Gender, male vs. female | 1.14 (0.83-1.57) | 0.41 | 1.19 (0.86-1.64) | 0.31 |
| Age, years | 1.04 (1.02-1.05) | **<0.01** | 1.04 (1.03-1.06) | **<0.01** |
| COPD, yes vs. no | 1.08 (0.78-1.49) | 0.65 | 1.08 (0.76-1.52) | 0.68 |
| Chronic Heart Failure, yes vs. no | 1.7 (1.03-2.81) | **0.04** | 1.21 (0.72-2.03) | 0.48 |
| PaO2/FiO2-ratio, kPa† | 0.95 (0.84-1.06) | 0.34 | 0.93 (0.82-1.04) | 0.21 |
| Respiratory Infection, yes vs. no | 0.92 (0.68-1.26) | 0.62 | 0.93 (0.66-1.31) | 0.68 |
| Septic Shock, yes vs. no | 1.69 (1.18-2.41) | **<0.01** | 1.42 (0.95-2.11) | 0.08 |
| Bilirubin, mmol/L† | 1.19 (0.94-1.52) | 0.16 | 1.14 (0.89-1.46) | 0.3 |
| KDIGO-score >= 2, yes vs. no | 1.69 (1.22-2.35) | **<0.01** | 1.23 (0.86-1.75) | 0.27 |

*Syndecan-1 was analyzed as a continuous variable, and the HR associated with an increase from the 25^th^ to the 75^th^ percentile (21.0 – 118.4 ng/ml) is presented. † Modelled using a restricted cubic spline with 2 knots. COPD = Chronic Obstructive Pulmonary Disease, KDIGO-score =Kidney Disease, Improving Global Outcomes-score of acute kidney injury. 95 % CI = 95 % Confidence Interval

**Table S16** Association of soluble Thrombomodulin (sTM) as a continuous variable with 30-day all-cause mortality in mechanically ventilated patients in the intensive care unit – Cox-regression (n = 459)

|  | **30-day all-cause mortality** | | | |
| --- | --- | --- | --- | --- |
|  | *Univariable* | | *Multivariable* | |
| *Predictor* | *Hazard Ratio (95% CI)* | *p* | *Hazard Ratio (95% CI)* | *p* |
| sTM, 25th vs. 75th percentile* | 2.23 (1.69-2.96) | **<0.01** | 1.92 (1.35-2.74) | **<0.01** |
| Gender, male vs. female | 1.14 (0.83-1.57) | 0.41 | 1.17 (0.85-1.62) | 0.34 |
| Age, years | 1.04 (1.02-1.05) | **<0.01** | 1.04 (1.02-1.05) | **<0.01** |
| COPD, yes vs. no | 1.08 (0.78-1.49) | 0.65 | 1.18 (0.84-1.66) | 0.35 |
| Chronic Heart Failure, yes vs. no | 1.7 (1.03-2.81) | **0.04** | 1.21 (0.72-2.03) | 0.48 |
| PaO2/FiO2-ratio, kPa† | 0.95 (0.84-1.06) | 0.34 | 0.94 (0.83-1.05) | 0.29 |
| Respiratory Infection, yes vs. no | 0.92 (0.68-1.26) | 0.62 | 0.97 (0.69-1.36) | 0.86 |
| Septic Shock, yes vs. no | 1.69 (1.18-2.41) | **<0.01** | 1.38 (0.93-2.06) | 0.11 |
| Bilirubin, mmol/L† | 1.19 (0.94-1.52) | 0.16 | 1.16 (0.91-1.48) | 0.23 |
| KDIGO-score >= 2, yes vs. no | 1.69 (1.22-2.35) | **<0.01** | 0.97 (0.66-1.43) | 0.89 |

* sTM was analyzed as a continuous variable, and the HR associated with an increase from the 25^th^ to the 75^th^ percentile (5.0 – 18.0 ng/ml) is presented. †Modelled using a restricted cubic spline with 2 knots. COPD = Chronic Obstructive Pulmonary Disease, KDIGO-score =Kidney Disease, Improving Global Outcomes-score of acute kidney injury. 95 % CI = 95 % Confidence Interval.

**Table S17** Association of Platelet Endothelial Cell Adhesion Molecule-1 (PECAM-1) as a continuous variable with 30-day all-cause mortality in mechanically ventilated patients in the intensive care unit – Cox-regression (n = 459)

|  | **30-day all-cause mortality** | | | |
| --- | --- | --- | --- | --- |
|  | *Univariable* | | *Multivariable* | |
| *Predictor* | *Hazard Ratio (95% CI)* | *p* | *Hazard Ratio (95% CI)* | *p* |
| PECAM-1, 25th vs. 75th percentile* | 1.53 (1.23-1.9) | **<0.01** | 1.42 (1.12-1.81) | **<0.01** |
| Gender, male vs. female | 1.14 (0.83-1.57) | 0.41 | 1.22 (0.88-1.69) | 0.23 |
| Age, years | 1.04 (1.02-1.05) | **<0.01** | 1.04 (1.03-1.06) | **<0.01** |
| COPD, yes vs. no | 1.08 (0.78-1.49) | 0.65 | 1.07 (0.76-1.5) | 0.71 |
| Chronic Heart Failure, yes vs. no | 1.7 (1.03-2.81) | **0.04** | 1.16 (0.69-1.96) | 0.57 |
| PaO2/FiO2-ratio, kPa† | 0.95 (0.84-1.06) | 0.34 | 0.94 (0.83-1.05) | 0.28 |
| Respiratory Infection, yes vs. no | 0.92 (0.68-1.26) | 0.62 | 0.94 (0.67-1.31) | 0.7 |
| Septic Shock, yes vs. no | 1.69 (1.18-2.41) | **<0.01** | 1.44 (0.97-2.14) | 0.07 |
| Bilirubin, mmol/L† | 1.19 (0.94-1.52) | 0.16 | 1.15 (0.9-1.47) | 0.27 |
| KDIGO-score >= 2, yes vs. no | 1.69 (1.22-2.35) | **<0.01** | 1.22 (0.85-1.73) | 0.28 |

* PECAM-1 was analyzed as a continuous variable, and the HR associated with an increase from the 25^th^ to the 75^th^ percentile (11.1 – 14.6 ng/ml) is presented. †Modelled using a restricted cubic spline with 2 knots. COPD = Chronic Obstructive Pulmonary Disease, KDIGO-score =Kidney Disease, Improving Global Outcomes-score of acute kidney injury. 95 % CI = 95 % Confidence Interval.

**Table S18** Association of Syndecan-1 as a continuous variable with PaO2/FiO2-ratio in mechanically ventilated patients on the first day of ICU-admission in all patients and on last measurement in patients dying before ICU-day five – linear regression.

|  | **PaO2/FiO2-ratio on first day of ICU-admission*** | | | | **Last PaO2/FiO2-ratio in non-survivors**† | | | |
| --- | --- | --- | --- | --- | --- | --- | --- | --- |
|  | *Univariable* | | *Multivariable* | | *Univariable* | | *Multivariable* | |
| *Predictor* | *Estimate (95% CI)* | *p* | *Estimate (95% CI)* | *p* | *Estimate (95% CI)* | *p* | *Estimtate (95% CI)* | *p* |
| Syndecan-1, 25th vs. 75th percentile‡ | 0.95 (0.89-1) | 0.07 | 0.94 (0.89-1) | 0.05 | 1.03 (0.87-1.21) | 0.76 | 1.03 (0.87-1.23) | 0.69 |
| Age, years | 1 (0.99-1) | **0.01** | 1 (0.99-1) | **0.01** | 1 (0.98-1.01) | 0.85 | 1 (0.98-1.02) | 0.94 |
| COPD, yes vs. no | 1.07 (0.97-1.17) | 0.18 | 1.13 (1.03-1.24) | **0.01** | 1 (0.76-1.33) | 0.98 | 1.06 (0.8-1.4) | 0.68 |
| Respiratory Infection, yes vs. no | 0.79 (0.72-0.86) | **<0.01** | 0.77 (0.7-0.84) | **<0.01** | 0.69 (0.53-0.9) | **0.01** | 0.67 (0.5-0.88) | **0.01** |
| Shock, yes vs. no | 0.95 (0.86-1.06) | 0.4 | 0.91 (0.82-1.02) | 0.1 | 0.93 (0.7-1.23) | 0.61 | 0.85 (0.63-1.15) | 0.29 |

*n = 459, † n = 62, ‡ Syndecan-1 was analyzed as a continuous variable, and the change in PaO2/FiO2-ratio associated with an increase from the 25^th^ to the 75^th^ percentile (21.0 – 118.4 ng/ml) is presented. COPD = Chronic Obstructive Pulmonary Disease. Shock = Need for vasopressor & lactate >= 2 at ICU-admission regardless of etiology. 95% CI = 95% confidence interval.

**Table S19** Association of soluble Thrombomodulin (sTM) as a continuous variable with PaO2/FiO2-ratio in mechanically ventilated patients on the first day of ICU-admission in all patients and on last measurement in patients dying before ICU-day five – linear regression.

|  | **PaO2/FiO2-ratio on first day of ICU-admission*** | | | | **Last PaO2/FiO2-ratio in non-survivors**† | | | |
| --- | --- | --- | --- | --- | --- | --- | --- | --- |
|  | *Univariable* | | *Multivariable* | | *Univariable* | | *Multivariable* | |
| *Predictor* | *Estimate (95% CI)* | *p* | *Estimate (95% CI)* | *p* | *Estimate (95% CI)* | *p* | *Estimtate (95% CI)* | *p* |
| sTM, 25th vs. 75th percentile‡ | 0.87 (0.81-0.95) | **<0.01** | 0.88 (0.81-0.95) | **<0.01** | 1.13 (0.86-1.48) | 0.37 | 1.18 (0.9-1.55) | 0.21 |
| Age, years | 1 (0.99-1) | **0.01** | 1 (0.99-1) | **0.02** | 1 (0.98-1.01) | 0.85 | 1 (0.99-1.02) | 0.79 |
| COPD, yes vs. no | 1.07 (0.97-1.17) | 0.18 | 1.11 (1.01-1.22) | **0.02** | 1 (0.76-1.33) | 0.98 | 1.05 (0.8-1.37) | 0.75 |
| Respiratory Infection, yes vs. no | 0.79 (0.72-0.86) | **<0.01** | 0.76 (0.69-0.83) | **<0.01** | 0.69 (0.53-0.9) | **0.01** | 0.66 (0.5-0.87) | **<0.01** |
| Shock, yes vs. no | 0.95 (0.86-1.06) | 0.4 | 0.93 (0.84-1.04) | 0.19 | 0.93 (0.7-1.23) | 0.61 | 0.84 (0.62-1.12) | 0.24 |

*n = 459, † n = 62, ‡ sTM was analyzed as a continuous variable, and the change in PaO2/FiO2-ratio associated with an increase from the 25^th^ to the 75^th^ percentile (5.0 – 18.0 ng/ml) is presented. COPD = Chronic Obstructive Pulmonary Disease. Shock = Need for vasopressor & lactate >= 2 at ICU-admission regardless of etiology. 95% CI = 95% confidence interval.

**Table S20** Association of Platelet Endothelial Cell Adhesion Molecule-1 (PECAM-1) as a continuous variable with PaO2/FiO2-ratio in mechanically ventilated patients on the first day of ICU-admission in all patients and on last measurement in patients dying before ICU-day five – linear regression.

|  | **PaO2/FiO2-ratio on first day of ICU-admission*** | | | | **Last PaO2/FiO2-ratio in non-survivors**† | | | |
| --- | --- | --- | --- | --- | --- | --- | --- | --- |
|  | *Univariable* | | *Multivariable* | | *Univariable* | | *Multivariable* | |
| *Predictor* | *Estimate (95% CI)* | *p* | *Estimate (95% CI)* | *p* | *Estimate (95% CI)* | *p* | *Estimtate (95% CI)* | *p* |
| PECAM-1, 25th vs. 75th percentile‡ | 0.91 (0.86-0.97) | **<0.01** | 0.91 (0.86-0.97) | **<0.01** | 1.11 (0.89-1.38) | 0.34 | 1.21 (0.97-1.51) | 0.09 |
| Age, years | 1 (0.99-1) | **0.01** | 1 (0.99-1) | **0.01** | 1 (0.98-1.01) | 0.85 | 1 (0.99-1.02) | 0.61 |
| COPD, yes vs. no | 1.07 (0.97-1.17) | 0.18 | 1.14 (1.04-1.25) | **0.01** | 1 (0.76-1.33) | 0.98 | 1.08 (0.83-1.42) | 0.57 |
| Respiratory Infection, yes vs. no | 0.79 (0.72-0.86) | **<0.01** | 0.77 (0.7-0.84) | **<0.01** | 0.69 (0.53-0.9) | **0.01** | 0.63 (0.48-0.83) | **<0.01** |
| Shock, yes vs. no | 0.95 (0.86-1.06) | 0.4 | 0.91 (0.82-1.02) | 0.09 | 0.93 (0.7-1.23) | 0.61 | 0.85 (0.63-1.13) | 0.26 |

*n = 459, † n = 62, ‡ PECAM-1 was analyzed as a continuous variable, and the change in PaO2/FiO2-ratio associated with an increase from the 25^th^ to the 75^th^ percentile (11.1 – 14.6 ng/ml) is presented. COPD = Chronic Obstructive Pulmonary Disease. Shock = Need for vasopressor & lactate >= 2 at ICU-admission regardless of etiology. 95% CI = 95% confidence interval

**Table S21** Association of Syndecan-1 as a continuous variable with oxygenation index in mechanically ventilated patients on the first day of ICU-admission in all patients and on last measurement in patients dying before ICU-day five – linear regression.

|  | **Oxygenation index on first day of ICU-stay*** | | | | **Last oxygenation index measured in non-survivors**† | | | |
| --- | --- | --- | --- | --- | --- | --- | --- | --- |
|  | *Univariable* | | *Multivariable* | | *Univariable* | | *Multivariable* | |
| *Predictor* | *Estimate (95% CI)* | *p* | *Estimate (95% CI)* | *p* | *Estimate (95% CI)* | *p* | *Estimtate (95% CI)* | *p* |
| Syndecan-1, 25th vs. 75th percentile‡ | 1.08 (0.99-1.18) | 0.07 | 1.06 (0.98-1.16) | 0.15 | 1.04 (0.8-1.36) | 0.74 | 1.04 (0.8-1.36) | 0.77 |
| Age, years | 1 (0.99-1.01) | 1 | 1 (1-1.01) | 0.87 | 1 (0.97-1.02) | 0.68 | 0.99 (0.97-1.02) | 0.51 |
| COPD, yes vs. no | 0.85 (0.74-0.98) | **0.03** | 0.82 (0.71-0.94) | **0.01** | 1.05 (0.65-1.67) | 0.85 | 1.02 (0.65-1.62) | 0.92 |
| Respiratory Infection, yes vs. no | 1.31 (1.15-1.5) | **<0.01** | 1.42 (1.24-1.62) | **<0.01** | 1.86 (1.21-2.86) | **0.01** | 1.92 (1.21-3.03) | **0.01** |
| Shock, yes vs. no | 1.17 (1-1.38) | 0.05 | 1.24 (1.05-1.45) | **0.01** | 1.01 (0.65-1.58) | 0.95 | 1.05 (0.66-1.66) | 0.84 |

*n = 459, † n = 49, ‡ Syndecan-1 was analyzed as a continuous variable, and the change in PaO2/FiO2-ratio associated with an increase from the 25^th^ to the 75^th^ percentile (21.0 – 118.4 ng/ml) is presented. COPD = Chronic Obstructive Pulmonary Disease. Shock = Need for vasopressor & lactate >= 2 at ICU-admission regardless of etiology. 95% CI = 95% confidence interval.

**Table S22** Association of soluble Thrombomodulin (sTM) as a continuous variable with oxygenation index in mechanically ventilated patients on the first day of ICU-admission in all patients and on last measurement in patients dying before ICU-day five – linear regression.

|  | **Oxygenation index on first day of ICU-stay*** | | | | **Last oxygenation index measured in non-survivors**† | | | |
| --- | --- | --- | --- | --- | --- | --- | --- | --- |
|  | *Univariable* | | *Multivariable* | | *Univariable* | | *Multivariable* | |
| *Predictor* | *Estimate (95% CI)* | *p* | *Estimate (95% CI)* | *p* | *Estimate (95% CI)* | *p* | *Estimtate (95% CI)* | *p* |
| sTM, 25th vs. 75th percentile‡ | 1.27 (1.12-1.44) | **<0.01** | 1.25 (1.1-1.41) | **<0.01** | 1.02 (0.66-1.56) | 0.94 | 1.02 (0.66-1.58) | 0.92 |
| Age, years | 1 (0.99-1.01) | 1 | 1 (0.99-1) | 0.77 | 1 (0.97-1.02) | 0.68 | 0.99 (0.97-1.02) | 0.51 |
| COPD, yes vs. No | 0.85 (0.74-0.98) | **0.03** | 0.84 (0.73-0.97) | **0.02** | 1.05 (0.65-1.67) | 0.85 | 1.01 (0.64-1.59) | 0.96 |
| Respiratory Infection, yes vs. no | 1.31 (1.15-1.5) | **<0.01** | 1.43 (1.25-1.64) | **<0.01** | 1.86 (1.21-2.86) | **0.01** | 1.92 (1.22-3.04) | **0.01** |
| Shock, yes vs. No | 1.17 (1-1.38) | 0.05 | 1.18 (1.01-1.39) | **0.04** | 1.01 (0.65-1.58) | 0.95 | 1.06 (0.67-1.67) | 0.81 |

*n = 459, † n = 49, ‡ sTM was analyzed as a continuous variable, and the change in PaO2/FiO2-ratio associated with an increase from the 25^th^ to the 75^th^ percentile (5.0 – 18.0 ng/ml) is presented. COPD = Chronic Obstructive Pulmonary Disease. Shock = Need for vasopressor & lactate >= 2 at ICU-admission regardless of etiology. 95% CI = 95% confidence interval.

**Table S23** Association of Platelet Endothelial Cell Adhesion Molecule-1 (PECAM-1) as a continuous variable with oxygenation index in mechanically ventilated patients on the first day of ICU-admission in all patients and on last measurement in patients dying before ICU-day five – linear regression.

|  | **Oxygenation index on first day of ICU-stay*** | | | | **Last oxygenation index measured in non-survivors**† | | | |
| --- | --- | --- | --- | --- | --- | --- | --- | --- |
|  | *Univariable* | | *Multivariable* | | *Univariable* | | *Multivariable* | |
| *Predictor* | *Estimate (95% CI)* | *p* | *Estimate (95% CI)* | *p* | *Estimate (95% CI)* | *p* | *Estimtate (95% CI)* | *p* |
| PECAM-1, 25th vs. 75th percentile‡ | 1.15 (1.05-1.25) | **<0.01** | 1.13 (1.03-1.23) | **0.01** | 1.05 (0.74-1.5) | 0.78 | 0.9 (0.63-1.31) | 0.59 |
| Age, years | 1 (0.99-1.01) | 1 | 1 (1-1.01) | 0.89 | 1 (0.97-1.02) | 0.68 | 0.99 (0.97-1.01) | 0.41 |
| COPD, yes vs. No | 0.85 (0.74-0.98) | **0.03** | 0.81 (0.7-0.93) | **<0.01** | 1.05 (0.65-1.67) | 0.85 | 0.99 (0.62-1.56) | 0.95 |
| Respiratory Infection, yes vs. no | 1.31 (1.15-1.5) | **<0.01** | 1.41 (1.23-1.61) | **<0.01** | 1.86 (1.21-2.86) | **0.01** | 1.99 (1.24-3.19) | **0.01** |
| Shock, yes vs. No | 1.17 (1-1.38) | 0.05 | 1.23 (1.05-1.44) | **0.01** | 1.01 (0.65-1.58) | 0.95 | 1.06 (0.67-1.67) | 0.81 |

*n = 459, † n = 49, ‡ PECAM-1 was analyzed as a continuous variable, and the change in PaO2/FiO2-ratio associated with an increase from the 25^th^ to the 75^th^ percentile (11.1 – 14.6 ng/ml) is presented. COPD = Chronic Obstructive Pulmonary Disease. Shock = Need for vasopressor & lactate >= 2 at ICU-admission regardless of etiology. 95% CI = 95% confidence interval

**Table S24** Association of Syndecan-1 as a continuous variable with ventilatory ratio in mechanically ventilated patients on the first day of ICU-admission in all patients and on last measurement in patients dying before ICU-day five – linear regression.

|  | **Ventilatory ratio on first day of ICU-stay*** | | | | **Last ventilatory ratio measured in non-survivors**† | | | |
| --- | --- | --- | --- | --- | --- | --- | --- | --- |
|  | *Univariable* | | *Multivariable* | | *Univariable* | | *Multivariable* | |
| *Predictor* | *Estimate (95% CI)* | *p* | *Estimate (95% CI)* | *p* | *Estimate (95% CI)* | *p* | *Estimtate (95% CI)* | *p* |
| Syndecan-1, 25th vs. 75th percentile‡ | 1.07 (0.99-1.15) | 0.07 | 1.06 (0.99-1.14) | 0.1 | 1.14 (0.93-1.39) | 0.19 | 1.11 (0.9-1.36) | 0.31 |
| Age, years | 1 (0.99-1) | 0.2 | 1 (0.99-1) | 0.06 | 1 (0.98-1.01) | 0.67 | 1 (0.98-1.02) | 0.97 |
| COPD, yes vs. No | 1.16 (1.05-1.28) | **<0.01** | 1.15 (1.04-1.28) | **0.01** | 0.68 (0.45-1.04) | 0.07 | 0.76 (0.47-1.22) | 0.23 |
| Respiratory Infection, yes vs. no | 1.19 (1.09-1.31) | **<0.01** | 1.21 (1.09-1.33) | **<0.01** | 1.43 (0.96-2.13) | 0.08 | 1.3 (0.84-2.02) | 0.22 |
| Shock, yes vs. no | 1.07 (0.95-1.19) | 0.26 | 1.11 (0.99-1.25) | 0.07 | 1.05 (0.72-1.53) | 0.78 | 1.06 (0.73-1.53) | 0.75 |

*n = 459, † n = 24, ‡ Syndecan-1 was analyzed as a continuous variable, and the change in PaO2/FiO2-ratio associated with an increase from the 25^th^ to the 75^th^ percentile (21.0 – 118.4 ng/ml) is presented. COPD = Chronic Obstructive Pulmonary Disease. Shock = Need for vasopressor & lactate >= 2 at ICU-admission regardless of etiology. 95% CI = 95% confidence interval.

**Table S25** Association of soluble Thrombomodulin (sTM) as a continuous variable with ventilatory ratio in mechanically ventilated patients on the first day of ICU-admission in all patients and on last measurement in patients dying before ICU-day five – linear regression.

|  | **Ventilatory ratio on first day of ICU-stay*** | | | | **Last ventilatory ratio measured in non-survivors**† | | | |
| --- | --- | --- | --- | --- | --- | --- | --- | --- |
|  | *Univariable* | | *Multivariable* | | *Univariable* | | *Multivariable* | |
| *Predictor* | *Estimate (95% CI)* | *p* | *Estimate (95% CI)* | *p* | *Estimate (95% CI)* | *p* | *Estimtate (95% CI)* | *p* |
| sTM, 25th vs. 75th percentile‡ | 1.12 (1.01-1.25) | **0.03** | 1.25 (1.1-1.41) | **<0.01** | 1.23 (0.84-1.79) | 0.27 | 1.31 (0.87-1.98) | 0.19 |
| Age, years | 1 (0.99-1) | 0.2 | 1 (0.99-1) | 0.77 | 1 (0.98-1.01) | 0.67 | 1 (0.98-1.03) | 0.66 |
| COPD, yes vs. no | 1.16 (1.05-1.28) | **<0.01** | 0.84 (0.73-0.97) | **0.02** | 0.68 (0.45-1.04) | 0.07 | 0.71 (0.44-1.14) | 0.15 |
| Respiratory Infection, yes vs. no | 1.19 (1.09-1.31) | **<0.01** | 1.43 (1.25-1.64) | **<0.01** | 1.43 (0.96-2.13) | 0.08 | 1.32 (0.86-2.03) | 0.19 |
| Shock, yes vs. no | 1.07 (0.95-1.19) | 0.26 | 1.18 (1.01-1.39) | **0.04** | 1.05 (0.72-1.53) | 0.78 | 1.05 (0.73-1.5) | 0.8 |

*n = 459, † n = 24, ‡ sTM was analyzed as a continuous variable, and the change in PaO2/FiO2-ratio associated with an increase from the 25^th^ to the 75^th^ percentile (5.0 – 18.0 ng/ml) is presented. COPD = Chronic Obstructive Pulmonary Disease. Shock = Need for vasopressor & lactate >= 2 at ICU-admission regardless of etiology. 95% CI = 95% confidence interval.

**Table S26** Association of Platelet Endothelial Cell Adhesion Molecule-1 (PECAM-1) as a continuous variable with ventilatory ratio in mechanically ventilated patients on the first day of ICU-admission in all patients and on last measurement in patients dying before ICU-day five – linear regression.

|  | **Ventilatory ratio on first day of ICU-stay*** | | | | **Last ventilatory ratio measured in non-survivors**† | | | |
| --- | --- | --- | --- | --- | --- | --- | --- | --- |
|  | *Univariable* | | *Multivariable* | | *Univariable* | | *Multivariable* | |
| *Predictor* | *Estimate (95% CI)* | *p* | *Estimate (95% CI)* | *p* | *Estimate (95% CI)* | *p* | *Estimtate (95% CI)* | *p* |
| PECAM-1, 25th vs. 75th percentile‡ | 1.09 (1-1.18) | **0.04** | 1.07 (0.99-1.16) | 0.09 | 1.35 (0.97-1.89) | 0.07 | 1.32 (0.92-1.9) | 0.13 |
| Age, years | 1 (0.99-1) | 0.2 | 1 (0.99-1) | 0.05 | 1 (0.98-1.01) | 0.67 | 1 (0.98-1.02) | 0.71 |
| COPD, yes vs. no | 1.16 (1.05-1.28) | **<0.01** | 1.15 (1.04-1.27) | **0.01** | 0.68 (0.45-1.04) | 0.07 | 0.72 (0.45-1.14) | 0.15 |
| Respiratory Infection, yes vs. no | 1.19 (1.09-1.31) | **<0.01** | 1.2 (1.09-1.32) | **<0.01** | 1.43 (0.96-2.13) | 0.08 | 1.23 (0.79-1.9) | 0.34 |
| Shock, yes vs. no | 1.07 (0.95-1.19) | 0.26 | 1.12 (1-1.26) | 0.06 | 1.05 (0.72-1.53) | 0.78 | 1.06 (0.75-1.52) | 0.72 |

*n = 459, † n = 24, ‡ PECAM-1 was analyzed as a continuous variable, and the change in PaO2/FiO2-ratio associated with an increase from the 25^th^ to the 75^th^ percentile (11.1 – 14.6 ng/ml) is presented. COPD = Chronic Obstructive Pulmonary Disease. Shock = Need for vasopressor & lactate >= 2 at ICU-admission regardless of etiology. 95% CI = 95% confidence interval
